# Supplementary material for: Health inequity: Possibilities of initiating pulmonary telerehabilitation programs for adults with chronic obstructive pulmonary disorders in conflict and low-resourced areas; A mixed-method phenomenological study
Source: PLoS One. 2025 May 29;20(5):e0324624. doi: 10.1371/journal.pone.0324624 (PMC12121761; doi:10.1371/journal.pone.0324624)
Supplement: S1 Table — (DOCX) [file pone.0324624.s001.docx]

Additional file 1- S1-S5 tables- Health inequity: possibility of initiating Pulmonary Telerehabilitation Program for Adults with Chronic Obstructive Pulmonary Disorders in conflict and low-resourced areas; A mixed-methods phenomenological study

S1 Table: Results of document analysis and SD questionnaire

| **Document analysis results** | | | | **Questionnaire results** | | | |
| --- | --- | --- | --- | --- | --- | --- | --- |
| Factor | Variable | N (70) | % | Factor | Variable | N (13) | % |
| Gender | Male | 68 |  | Gender | Male | 13 | 100% |
|  | Female | 2 |  | Age | mean± SD | 66,07±9.84 |  |
| Admission criteria | Dyspnea | 53 | 75.7% | MBI | mean± SD | 35.2±13.35 |  |
|  | Coughing | 50 | 71.4% | Years since being diagnosed with COPD | mean± SD | 6.88±7,52 |  |
|  | Sputum | 20 | 28.5% | Social status | Married | 12 | 92.3% |
|  | SPO2 | 63 | 90% |  | Divorced | 1 | 7.7% |
|  | Heart rate | 21 | 30% | Education level | Not educated | 4 | 30.73% |
|  | Respiratory rate | 36 | 51.4% |  | Preliminary | 4 | 30.73% |
|  | Cyanosis | 6 | 8.6% |  | Secondary | 4 | 30.73% |
|  | High temperature | 30 | 42.8% |  | Undergraduate | 1 | 7.7% |
|  | Auscultation | 28 | 40% | Working | Yes | 1 | 7.7% |
| Comorbidities | Hypertension | 32 | 45.7% | Comorbidities | Current smoking | 5 | 38.5% |
|  | Type 2 diabetes | 22 | 31.4% |  | Diabetes | 8 | 61.5% |
|  | Peripheral artery disease | 1 | 1.4% |  | Hypertension | 7 | 53.8% |
|  | Smoking | 20 | 28.6% |  | Vascular disorder | 0 | 0 |
|  | Congestive heart failure | 23 | 32.9% |  | Cardiac disorder | 6 | 46.2% |
|  | Difficult walking | Zero | - |  | Eye problems | 2 | 15.4% |
|  | Pneumonia | 13 | 18.6% |  | Walking difficulties | 10 | 76.9% |
|  | Lung cancer | 2 | 2.9% |  | Obesity | 5 | 38.5% |
|  | Obesity | 4 | 5.7% |  | Stroke | 4 | 30.8% |
|  | Depression | 2 | 2.9% |  | Stress and anxiety | 8 | 61.5% |
|  | Anxiety | Zero | - |  | Previous surgeries | 8 | 61.5% |
|  | Covid-19 | 9 | 12.9% |  |  |  |  |
|  | Stroke | 2 | 2.9% |  |  |  |  |
| Investigations | Chest X-ray | 50 | 71.4% |  |  |  |  |
|  | CT scan | 8 | 11.4% |  |  |  |  |
|  | Spirometry | Zero | - |  |  |  |  |
|  | Bronchoscopy | Zero | - |  |  |  |  |
|  | ABGs | 51 | 72.8% |  |  |  |  |
|  | CBC | 64 | 91.4% |  |  |  |  |
|  | ECG | 53 | 75.7% |  |  |  |  |
|  | Electrolyte | 51 | 72.8% |  |  |  |  |
| Medical intervention | O2 therapy | 33 | 47.1% |  |  |  |  |
|  | Antibiotics | 55 | 78.6% |  |  |  |  |
|  | Bronchodilators | 13 | 18.6% |  |  |  |  |
|  | Supplements1 | 59 | 84.3% |  |  |  |  |
|  | Antibiotics-H2 | 28 | 40% |  |  |  |  |
|  | Bronchodilators-H2 | 40 | 57.1% |  |  |  |  |
|  | Antispasmodic-H2 | 23 | 32.8% |  |  |  |  |
|  | Surgery | Zero | - |  |  |  |  |
|  | Referral to a specialist | Zero | - |  |  |  |  |
| Referred to PT | Written referral | 5 | 7.1% | Current COPD symptoms | Dyspnea | 12 | 92.3% |
|  | Oral referral | 7 | 10% |  | Coughing | 9 | 69.2% |
|  | None | 58 | 82.8% |  | Sputum | 9 | 69.2% |
|  |  |  |  |  | Fatigue | 11 | 84.6% |
|  |  |  |  |  | Inability to do ADL | 11 | 84.6% |
| Receive in-hospital PT | Yes | 22 | 31.4% | Receive in-hospital PT | Yes | 13 | 100% |
| Discharge criteria | Dyspnea relief | 21 | 30% | Post discharge management | medications | 13 | 100% |
|  | Improved breathing pattern | 5 | 7.1% |  | follow-up with doctors | 6 | 46.2% |
|  | Chest clearance | 19 | 27.1% |  | PT | 3 | 23.1% |
|  | Conscious level | 11 | 15.7% |  |  |  |  |
|  | Improved ADL | 1 | 1.4% |  |  |  |  |
|  | Enhanced SPO2 | 36 | 51.4% |  |  |  |  |
|  | Normalized resp. rate | 14 | 20% |  |  |  |  |
|  | Normalized HR | 17 | 24.3% |  |  |  |  |
|  | Normalized temperature | 21 | 30% |  |  |  |  |
|  | Improved air entry | 15 | 21.4% |  |  |  |  |
|  | Improved lung function | 1 | 1.4% |  |  |  |  |
