# Supplementary material for: Health inequity: Possibilities of initiating pulmonary telerehabilitation programs for adults with chronic obstructive pulmonary disorders in conflict and low-resourced areas; A mixed-method phenomenological study
Source: PLoS One. 2025 May 29;20(5):e0324624. doi: 10.1371/journal.pone.0324624 (PMC12121761; doi:10.1371/journal.pone.0324624)
Supplement: S2 Table — (DOCX) [file pone.0324624.s002.docx]

Additional file 1- S1-S5 tables- Health inequity: possibility of initiating Pulmonary Telerehabilitation Program for Adults with Chronic Obstructive Pulmonary Disorders in conflict and low-resourced areas; A mixed-methods phenomenological study

S2 Table: Patients' experience with in-hospital PT, need for PR, and willingness to adopt PTR.

| **Factor** | **Variable** | **Mean ± SD** |
| --- | --- | --- |
| Patients’ experience with the received In-hospital physiotherapy | - I received a full explanation from the physiotherapist about my health. | 2.46 ± 1.561 |
|  | - I received a full explanation from the physiotherapist about the importance of physiotherapy to treat my condition. | 2.31 ± 1.377 |
|  | - I received suitable physiotherapy sessions at the hospital. | 2.62 ± 1.710 |
|  | - I felt comfortable having received physiotherapy services at the hospital. | 2.62 ± 1.660 |
|  | -I felt better after receiving physiotherapy services at the hospital. | 2.38 ± 1.609 |
|  | - I received the appropriate education to carry out a home exercises program (breathing, rest positions, and sputum clearance) | 2.69 ± 1.653 |
|  | - I received the appropriate education to use  therapeutic devices such as incentive spirometry | 2.46 ± 1.808 |
| **- Determine on a scale of 1-5 the satisfaction of physiotherapy services while you are in the hospital** | | **2.15 ± 1.573** |
| The need for physiotherapy services post- discharge from the hospital | -I need for physiotherapy services after discharge from the hospital | 4.31 ± 1.182 |
|  | -I am comfortable to continue learning about my illness through a physiotherapist | 4.15 ± 1.144 |
|  | - Receiving physiotherapy services will help me  overcome my symptoms | 4.08 ± 1.256 |
|  | -Receiving physiotherapy services will help me improve my lung health. | 4.08 ± 1.115 |
|  | -I would like to continue receiving physiotherapy service through a specialized physiotherapy center | 4.31 ± 1.182 |
|  | - I would like to continue receiving physiotherapy service through home visits | 4.54 ± 1.198 |
|  | - I would like to continue follow-up physiotherapy service through phone, mobile SMS, mobile application | 3.77 ± 1.481 |
|  | -I would like to receive physiotherapy service through a combination of center/hospital visits and home follow-up | 4.31 ± 1.182 |
| **Determine on a scale of 1-5 how much you need physiotherapy services after discharge from the hospital** | | **4.38 ± 1.193** |
| Willingness to receive telerehabilitation services through | -I have an idea about telerehabilitation services | 3.85 ± .987 |
|  | -I am willing to communicate with a physiotherapist through the mobile app | 3.23 ± 1.301 |
|  | -I am willing to communicate with a physiotherapist through SMS | 3.23 ± 1.013 |
|  | -I am willing to do a home physiotherapy program using therapeutic devices | 3.69 ± 1.182 |
|  | -I am willing to learn therapeutic exercises (breathing exercises) through videos sent by physiotherapists | 3.77 ± 1.013 |
|  | - I expect telerehabilitation services to help me overcome my symptoms. | 3.92 ± .954 |
|  | - I expect telerehabilitation services to help me improve lung health. | 3.85 ± 1.068 |
|  | - I would be happy if I could do a physiotherapy program at home | 4.31 ± 1.032 |
| **Determine on a scale of 1-5 your willingness to receive physiotherapy services using modern technology (mobile app, SMS, therapeutic devices, therapeutic exercise video)** | | **3.77 ± .832** |
