# Supplementary material for: Health inequity: Possibilities of initiating pulmonary telerehabilitation programs for adults with chronic obstructive pulmonary disorders in conflict and low-resourced areas; A mixed-method phenomenological study
Source: PLoS One. 2025 May 29;20(5):e0324624. doi: 10.1371/journal.pone.0324624 (PMC12121761; doi:10.1371/journal.pone.0324624)
Supplement: S3 Table — (DOCX) [file pone.0324624.s003.docx]

Additional file 1- S1-S5 tables- Health inequity: possibility of initiating Pulmonary Telerehabilitation Program for Adults with Chronic Obstructive Pulmonary Disorders in conflict and low-resourced areas; A mixed-methods phenomenological study

S3 Table: Thematic Framework 1-3: Challenges

| **Subthemes** | **Sample Quotes** | **Responses (N)** |
| --- | --- | --- |
| **Theme 1: Environmental challenges** | | |
| PTR is not a priority in MoH’s practice and plans | M1: “TR is part of the ministry's strategy in dealing with epidemics, emergencies and wars, even in the plans of 2020, they rejected it, because we cannot measure the system effectiveness. I am not against it but they do not consider it a priority." | 1 (+) |
| PT is underprioritized in MoH plans and practice | P1: “The institution itself does not recognize physiotherapy as a fundamental care, but always considering it as auxiliary, and this applies all-over the system of MoH, last year, we had four PT offices integrated into the in-patient, this year, we lost one of them for no reason. The vice minister, in any regular meeting has questioned the importance of PT and propose to terminate the outpatient services.”  M2:” There is no places to upgrade PT services in the plans of MoH; a new, building is launched these days for the internal medicine, it is an 8-floor building, and will be one of the largest health care institutions in Gaza, there is no space in it for PT. I was shocked when the hospital director, outlined this plan and informed us that we don’t want physiotherapy there. I am wondering, where is the physiotherapy in internal medicine Hospital?” | 5 (+) |
| The impacts of conflict lead to unavailability of resources | M1:” people can travel long distances for training and gaining knowledge, but we can’t.”  PT1:” In Palestine, people own smartphones, but we can’t build a modern rehabilitation program, why? Because we are isolated from the outside world.”  D4:” because of the Siege, we are not allowed to import all of these devices." | 4 (+) |
| The unavailability of resource, influenced the management of COPD | Lack of devices (Spirometer) (+5)  D3:” We are not using pulmonary function test in practice, because spirometer is not available in MoH hospitals. so, there is a drop in our diagnosis for patients with COPD, according to the guidelines there is no diagnosis for COPD without pulmonary function tests.”  Lack of financial support for patients: (+4)  D1: “most patients who come to the hospital have no alternatives, they are unable to pay or can’t buy medications, and COPD medications are expensive. Patients brought the cheap ones from the UNRWA, which causes several collateral damages and side effects as the arrhythmia and it doesn’t give the desired effects.”  Lack of infrastructure: (+3)  M1:” the MoH is unable to establish the outpatient chest PT due to its cost, it needs a place with specific standards; a special unit equipped, a qualified staff, equipment and tools including the spirometry, laptops and treadmill. These essentials are not available, this is always the obstacle between PT and the MoH, they always reply to us: The question is: who will provide these devices?” | 8 (+) |
| The heavy burden of COPD is exacerbated by emergencies | D3: “a very large number people in the street are affected by COPD, about 10% of the community are Asthma patients, and almost two- thirds of them are COPD, but not diagnosed. In 2021 during the Corona pandemic, the patients are kept away from hospitalization due to fear of infection, there are no beds to accommodate patients in the presence of a pandemic like the Corona. Most of the hospital beds have turned into Corona patients.”, “Such large number of patients becomes very reasonable to open the outpatient PT unit as you propose.” | 3 (+) |
| **Theme 2: Professional challenges** | | |
| Work overload for doctors & physiotherapists | D1:” due to high number and overloading, only severe cases are referred to PT”  D5:” because of the large numbers admitted cases to the hospital, and limited number of existing beds, we discharge patients early.”  P5: "We are few physiotherapists who work for chest patients, so we can’t document all our work for them, here is the problem.” | 8 (+) |
| Doctors underestimate the roles of PT. | D3: “unfortunately the lack of scientific knowledge about the importance of physiotherapy in different departments is why they neglecting its role.”  D2: “hospitalization without physiotherapy doesn't make sense. Their role for patients with COPD is very important especially for exacerbation, all of post-covid patients requires physiotherapy. But there is a need for agreement between doctors and physiotherapists, as long as the doctor is the leader of the team."  D4: “most doctors do not take into account the importance of physiotherapy in cases of COPD or other chest diseases, there is popular idea that all physiotherapist can do is to ask patients to move, say cough, cough, and perform percussion on his back and finish. I think they are not taking their role in chest department. Physiotherapy can achieve good outcomes parallel to medical treatment.”  P3: “the shortage of knowledge of the medical staff about the role of physiotherapy, especially for COPD patients, they see physiotherapy as a clearance technique.”  M2: “we are still struggling with the professional role of physiotherapy; where, when, how and so on ... and we understand that there is a problem to convince doctors about our role, it is unsatisfactory ...”  M1:” It’s strange that doctors have requested specific referral form for physiotherapy, they were the first people who didn't commit.”  P2: ”doctor will not give the physiotherapist a space to prove his work, unfortunately this is a general problem in MoH.” | 11 (+10\ -1) |
| Lack of referral criteria to PT | D1: “According to the severity of the patient, most of patients who are referred to physiotherapy are in the advance stage.”  P4: ”the amount of sputum present .. does it need physiotherapy? .. Oh right, it's up to the doctor himself, there are three doctors who refer to physiotherapy, and the rest I haven’t recognized they’re referring.”  P1: “The criteria of referral to physiotherapy are the sputum, doctors believe physiotherapy can help in sputum expectoration, they have no idea about other physiotherapy interventions such as aerobic exercises, psychological support, breathing exercises. They also refer patient tin he advanced stage.” | 9 (+) |
| Lack of professionals' efforts to enhance experience and collaboration | D4: “Physiotherapists themselves are not giving indications or awareness of what they can do for patients, it is very important to prove their proficiency and show the results of their work .. even doctor should enhance their practice such as detailed investigations of secretions. We need more professional practice.”  P3: “Honestly, I’ve never tried to explain to doctors my actual role in physiotherapy beyond secretion expectoration, or propose to help patients in other dimensions. We only discuss cases during the morning round.”  M1: “as the head of the physiotherapy department, I can say that we are introverted to ourselves; there are many qualified physiotherapists in the department, no one took the initiative; start discussion, give information session, arrange for workshop, or launch cooperation with doctors.” | 5 (+) |
| Lack of shared discharge decision | D4:” the discharge decision is made based on clinical data, investigation data and patients’ general condition, we sometimes consider if physiotherapy is completed.”  D1:” "The problem is that we can't keep the patient at hospital for just receiving physiotherapy, as long as the patient improves, will be discharged.”  M1: “We do not have any authority to keep the patient at the hospital to continue physiotherapy program, sometimes, we might discuss with the doctor and succeed to keep them a day or two after the medical intervention is completed.”  P1:” it is possible that doctors find the patient starting to improve, then decide to discharge him. Physiotherapy is not part of this discharge decision.” | 10 (+) |
| Improper PT documentation | D2: “20% of referral rate to physiotherapy is a very small percentage, I don’t think that it touches the reality, uh, there's a bug in this documentation that's I'm sure of it, 60-70% of cases are referred to physiotherapy, this is the actual percentage.”  P3: “Most of the time it’s oral referral, so it’s not documented.”  P1:“The documentation system f the hospital especially, the archive, lack of physiotherapy documents.”  P5:” We work with almost all chest patients admitted to the department, but we document for sever cases only such as Pulmonary Embolism and Plural Effusion.”  M2: “three important defects in the documentation system; the current system is very primitive, that does not reflect the amount of work that is being done frankly. The other problem is the oral referral by doctors are not documents, the third problem, is the physiotherapists themselves, they are not documenting their work.” | 9 (+) |
| Difficulties in modifying patients’ risk factors | D3: “The commitment of the patients in this device is very weak, we repeat it and repeat, but the patients need always someone to remind them for that.”  D1: “We classified patients as A, B, C & D, they don’t go to the hospital until they reached stages C, D and required oxygen therapy”  D2: “they are smokers, they buy cigarette and not buying medicines, this is the culture of the society .. most of them are in their 50-60 years, which means not everyone holding a mobile phone, not everyone is answering Messenger and WhatsApp or using mobile app.”  D4: “did he recurrently admitted to the hospital? Yes, because he has become exacerbated, what the reason is, because he is still smoking, he is exposed to dust due to occupation, and he still in his place. It is because their behaviour or lifestyle can’t be modified easily."  P2: “the patients are elderly, they can use the mobile more than I do, but will they use it for follow-ups? Do they have the patience for that .. our patients become proud that they have been smoking for 50 years.” | 6 (+) |
