# Supplementary material for: Health inequity: Possibilities of initiating pulmonary telerehabilitation programs for adults with chronic obstructive pulmonary disorders in conflict and low-resourced areas; A mixed-method phenomenological study
Source: PLoS One. 2025 May 29;20(5):e0324624. doi: 10.1371/journal.pone.0324624 (PMC12121761; doi:10.1371/journal.pone.0324624)
Supplement: S4 Table — (DOCX) [file pone.0324624.s004.docx]

Additional file 1- S1-S5 tables- Health inequity: possibility of initiating Pulmonary Telerehabilitation Program for Adults with Chronic Obstructive Pulmonary Disorders in conflict and low-resourced areas; A mixed-methods phenomenological study

S4 Table: Thematic Framework 2-3: Opportunities

| **Subthemes** | **Sample Quotes** | **Responses (N)** |
| --- | --- | --- |
| **Theme 3: Environmental opportunities** | | |
| Possibilities to adapt health policies in MoH to provide PR and PTR services | M1: "38 physiotherapy departments are operating in Gaza strip, 15 of them operating in the hospitals and 23 are operating outside hospitals, and they are doing the same work. Let’s work more wisely. The primary care clinics provide outpatient physiotherapy, I can buy this service from them. Why does duplication happen?", “We are partners with the NGOs, we unite and integrate to provide continuum of care for our patients.”  M2: "I can refer to a nearby primary care clinic within the MoH.”  D4: “In the future you can operate the National Center for management of COPD Patients, and you can partner with the MoH." | 3 (+) |
| The MoH values the significance of PR and PTR | M1:” We are in a country that need to establish a specialized physiotherapy unit for Cardiopulmonary, and the idea of PTR is very fascinating not only at the level of the ministry, but also at the level of the health service as a whole”  M2: “Uh, I place a lot of importance toward TR, but I want people to work with me."  D4: “"It's a very good idea; the goals, the outcomes, and the anticipated impacts of the project, all are good." | 3 (+) |
| Managers in MoH support professional development | M2: “collaboration with doctors, this is a point of great interest within the physiotherapy department. How much did I try to open channels between doctors and therapists? Currently, our discussions about cases, and we hope to organize other joint activities.” | 1(+) |
| **Theme 4: Professional opportunities** | | |
| The perceived need for the post discharge PR | D3: “physiotherapy should be completed in the outpatient or at home or at the nearest outpatient physiotherapy center, but establishing outpatient physiotherapy clinic especially for chest patients is an excellent positive improvement.”  D5: “I'm totally with the idea of launching a rehabilitation center for all COPD patients; it is very important to reduce the stress and anxiety associated with COPD, and reduce the disease severity.”  P2: “we need to establish a unit in the outpatient to provide physiotherapy and follow-ups for COPD cases”  P3: "Uh, of course there is a need, as you said the inpatient physiotherapy is not enough, so it is necessary to continue physiotherapy after discharge.”  M1: “we encounter a serious problem due to the deprivation of chest physiotherapy services.”  M2: “this is as a pioneering idea, and we are in need for pulmonary rehabilitation unit, I am 100% supportive of it.” | 8 (+) |
| Why PTR is essential for the management of COPD? | D3: “I think the patients themselves will feel difference in their management and follow-up, they will have the sense of not being neglected at home, which might reduce the rate of readmission. Psychologic distress will be decreased.”  D1: “In my opinion, it will benefit the patient and benefit the Ministry of Health itself.”  D2: “It is a very good idea to launch a way of communication with the patients through technology. But we should consider the cos.t”  D4: “it's a very wonderful and new idea about COPD rehabilitation in Gaza.  P4: “I think patients will benefit from the PTR, he will receive continuous treatment and education, this will enhance his psychologic status, and motivate him to do exercises.”  P2: “In my opinion, PTR would be very effective; we will keep in touch with the patients, provide follow-up for them, patients will receive continuous treatment.”  M2: “PTR is evolving worldwide, and is a very important subject, we had this experience during the Corona lockdown, we launched communication with our patients through WhatsApp, we noticed the effects on our patients, on physical and psychologic aspects. I think PTR is a requirement for our healthcare system.” | 9 (+) |
| Possibilities to enhance professional practice | Integrate spirometry measurement into routine practice: (+6)  D2:” spirometry is essential for diagnosis and follow-up for every patient with COPD."  D4:” Of course, before and after intervention, you have to perform the lung function test, and decide if there is improvement or not? Did the total lung capacity and the FEV1&FEV2 improved or not?”  It’s essential to refer every patient with COPD to physiotherapy (+5)  D1:” there should be a common agreement among chest doctors and physiotherapists, that all cases of COPD should be referred to physiotherapy,”  M1:” it is supposed that the admitted patients in the hospital all need physical therapy regardless of the nature of the intervention, unless contraindicated.”  D3: “You should start with whom is referring, of course the doctor, there must be some kind of awareness about physiotherapy roles, and encourage physiotherapists to adhere to guidelines. So, you can improve the referral rate.”  Creation of specific referral form to physiotherapy: (+2, -3)  P1:” Create a specific form for chest physiotherapy cases”  M2: "The problem is the multiplicity of forms that causes confusion for professionals, it means that frankly the unification is always better."  D2:” we don't want to increase the writing on the doctor because he writes a lot of boring stuff.”  D3: “we are ready to create a certain sheet or referral for physiotherapy”  M1:” It’s strange that doctors have requested specific referral form for physiotherapy, they were the first people who didn't commit.” | - |
| Possibilities to enhance interprofessional collaboration | P2: “Exactly, it is the responsibility of the department of Physiotherapy to enhance cooperation with doctors."  P3: “Honestly there is no discussion between us, only doctor refer patients, sometimes we can’t see the doctors, he came to the department in a hurry”  P4: “Yes, sometimes we discuss during the morning round, and upon the need for the case”  D2: “It is possible that we arrange joint workshops to discuss issues related physiotherapy profession, and their role with patients”  M2: “scientific days, training, and workshops should be open to everyone, about 60% of the physiotherapists are enrolled in training annually .. we should start from the university that teaches doctors, and include in their courses the importance of physiotherapy intervention, these courses should be given by physiotherapists.” | 9 (+7\-2) |
| Willingness to adopt PTR among HCPs | D3: “as a doctor working in the government and private sectors, I’m very interested in providing such a distinguished service. This is a distinction that gives me a higher degree in the health services I provide to patients, God willing, we will have time to help in this area.”  D4: “Excellent, new and even possible to include other cases, such as post-covid syndrome, bronchitis or lung fibrosis. It means that you have covered virtually all cases of lung disease through telerehabilitation."  D5: “we hope that this service will be available for the benefit of our patient."  P3:” "It is possible if it becomes a specialized unit, or a place where it is possible to provide awareness about PTR and education about the program."  P5: “Yes, if this system is established, and the devices are provided, possible to work in it." | 11 (+) |
