# Supplementary material for: Health inequity: Possibilities of initiating pulmonary telerehabilitation programs for adults with chronic obstructive pulmonary disorders in conflict and low-resourced areas; A mixed-method phenomenological study
Source: PLoS One. 2025 May 29;20(5):e0324624. doi: 10.1371/journal.pone.0324624 (PMC12121761; doi:10.1371/journal.pone.0324624)
Supplement: S5 Table — (DOCX) [file pone.0324624.s005.docx]

Additional file 1- S1-S5 tables- Health inequity: possibility of Initiating Pulmonary Telerehabilitation Program for Adults with Chronic Obstructive Pulmonary Disorders in conflict and low-resourced area; A mixed-methods phenomenological study

S5 Table: Thematic Framework 3-3: Characteristics of the intended PTR

| **Subthemes** | | **Sample Quotes** | **Responses (N)** |
| --- | --- | --- | --- |
| **Theme 5: component of the intended PTR** | | | |
| Using therapeutic devices is recommended | P2: “We gave patients incentive spirometer and OPEP devices, and it was effective.”  D2: “it was a great device as we used it for some COVID patients admitted to hospital, and we adopted it, the ball device (incentive spirometer) in particular." | | 5 (+) |
| Mobile app is a potential tool for PTR | P1: “about 90% of patients have smart phones, and I wish we could apply the TR system using it. Similar to the bank’s message system, I no longer go to the bank. “  P2: “If a device is connected to a mobile application, it will be better because the whole world is paying attention to the mobile app to shorten the time and effort.”  D3: “most of patients own mobiles, so it is possible to work on mobile apps, but it seems more possible that you provide therapeutic devices to the patients”  D1: “If the mobile app is included in this system, it's great, it means decreasing work load on the hospital and reducing the patient's visits to the hospital." | | 9 (+) |
| Visual and audio contents are preferred | D3: “Most of our patients do not read, it is possible to watch a video. They do not like to read and write.”  P3: “The mobile application provides something audio, and something visual is better." | | 2 (+) |
| Requirements to launch the PTR | Awareness (+3): D3: "Before establishing this system, you need to build knowledge about the value of establishing it before referring patients to it."  Training (+2): P2: “it requires training courses for physiotherapists”  Incentives (+2): D2: "Uh, there's an incentive for your patients, such as transportation means, food, drink, and medicine. If the government does that to make them committed and manage the disease level."  Ethical standards (+1): M2: “Of course, Ethical standards are very important for establishing this system”  Ruling legislations (+2): P1: “we need the legislation to identify rights and responsibilities of physiotherapy in this system because the profession exists on the scene along with other professions.”  Multidisciplinary team and Fund (+1): D4: “It's all from doctors, physiotherapists, nurses, engineers, ITs, the Internet, laptops ..… and requires funders."  Caregivers (+1): D1: “the patient must have a family member with him to help him accept the idea, and use the device.” | | --- |
| Hybrid PTR regime | D2: "It can be performed between the hospital and the mobile as a mixed program."  P4: “It is good to adopt the technology, but if it will last for long periods, it is better to dedicate once a month to a visit to the hospital.” | | 2 (+) |
