# Supplementary material for: Health inequity: Possibilities of initiating pulmonary telerehabilitation programs for adults with chronic obstructive pulmonary disorders in conflict and low-resourced areas; A mixed-method phenomenological study
Source: PLoS One. 2025 May 29;20(5):e0324624. doi: 10.1371/journal.pone.0324624 (PMC12121761; doi:10.1371/journal.pone.0324624)
Supplement: Attachment — Submitted filename: Reviewed PONE-D-24-30276_N. Mshunqane.pdf [file pone.0324624.s006.pdf]

PLOS ONE

Health Inequity; Possibilities of Initiating Pulmonary Telerehabilitation Programs for Adults with Chronic Obstructive Pulmonary Disorders in Conflict and Low-Resourced Areas; A mixed-methods Phenomenological Study

--Manuscript Draft--

**Manuscript Number:**	PONE-D-24-30276
**Article Type:**	Research Article
**Full Title:**	Health Inequity; Possibilities of Initiating Pulmonary Telerehabilitation Programs for Adults with Chronic Obstructive Pulmonary Disorders in Conflict and Low-Resourced Areas; A mixed-methods Phenomenological Study
**Short Title:**	Initiating Pulmonary telerehabilitation for COPD in conflict-low-resource areas: mixed-method research
**Corresponding Author:**	Suad Joma Ghaben, PhD<br>Universiti Kebangsaan Malaysia<br>Kuala Lumpur, MALAYSIA
**Keywords:**	Keywords: Health equity; Pulmonary; telerehabilitation; COPD
**Abstract:**	Background: The "triple problems of COPD"—underdiagnosis, underrecognition, and underdevelopment of pulmonary rehabilitation programs—are global. Pulmonary telerehabilitation (PTR) may solve these problems. This study examines the possibility of launching PTR programs for adults in an area of conflict and low resources.<br>Methods: A mixed methods convergent design was used to understand outpatient PR unavailability. The medical records of 70 patients with COPD in the MoH chest unit were analysed quantitatively. 13 patients who received in-hospital physiotherapy were questioned via questionnaire, and 12 healthcare professionals (HCPs) were interviewed.<br>Results: 70 patients with a mean age of 65.3±1.65 years and a mean hospital stay of 4.89±0.392 days were managed with no admission, PT referral, or discharge criteria. Patients reported needing outpatient PR of 4.38 ± 1.193 and a willingness to adopt of 3.77 ±.832. Interviews with HCP highlight the environmental and professional challenges, opportunities, and PTR traits. Quantitative and qualitative data triangulation led to the COPD Sequential Triad (CST) theory.<br>Conclusion: This study illuminates the national, institutional, and professional PTR development challenges in conflict and low resources, including the sequels of conflict, insufficient resources, and the absence of a structured healthcare system and clinical practice guidelines. Promoting health equity in such areas requires the international community and institutions to isolate the healthcare sector from conflicts and apply more effective strategies.
**Order of Authors:**	Suad Joma Ghaben, PhD
	Arimi Fitri Mat Ludin, Associate Professor
	Badr Elkholi, Master Student
	Reem Kullab, Bachelor
	Majd Al-Hour, Bachelor
	Devinder Kaur Ajit Singh, Professor
**Additional Information:**	
**Question**	**Response**
**Financial Disclosure**<br><br>Enter a financial disclosure statement that describes the sources of funding for the work included in this submission. Review the submission guidelines for detailed	The author(s) received no specific funding for this work.

Powered by Editorial Manager® and ProduXion Manager® from Aries Systems Corporation

requirements. View published research articles from *PLOS ONE* for specific examples.<br><br>This statement is required for submission and **will appear in the published article** if the submission is accepted. Please make sure it is accurate.<br><br>**Funded studies**<br>Enter a statement with the following details:<br>• Initials of the authors who received each award<br>• Grant numbers awarded to each author<br>• The full name of each funder<br>• URL of each funder website<br>• Did the sponsors or funders play any role in the study design, data collection and analysis, decision to publish, or preparation of the manuscript?<br><br>Did you receive funding for this work?	
**Competing Interests**<br><br>Use the instructions below to enter a competing interest statement for this submission. On behalf of all authors, disclose any competing interests that could be perceived to bias this work—acknowledging all financial support and any other relevant financial or non-financial competing interests.<br><br>This statement is required for submission and **will appear in the published article** if the submission is accepted. Please make sure it is accurate and that any funding sources listed in your Funding Information later in the submission form are also declared in your Financial Disclosure statement.<br><br>View published research articles from *PLOS ONE* for specific examples.	The authors have declared that no competing interests exist.

Powered by Editorial Manager® and ProduXion Manager® from Aries Systems Corporation

**NO authors have competing interests**<br><br>Enter: *The authors have declared that no competing interests exist.*<br><br>**Authors with competing interests**<br><br>Enter competing interest details beginning with this statement:<br><br>*I have read the journal's policy and the authors of this manuscript have the following competing interests: [insert competing interests here]*<br><br>* typeset	
**Ethics Statement**<br><br>Enter an ethics statement for this submission. This statement is required if the study involved:<br><br>• Human participants<br>• Human specimens or tissue<br>• Vertebrate animals or cephalopods<br>• Vertebrate embryos or tissues<br>• Field research<br><br>Write "N/A" if the submission does not require an ethics statement.<br><br>General guidance is provided below. Consult the submission guidelines for detailed instructions. **Make sure that all information entered here is included in the Methods section of the manuscript.**	*The MoH committee approved the research protocol. document no. 993123.<br>*Written informed consent was obtained from the study participants.

Powered by Editorial Manager® and ProduXion Manager® from Aries Systems Corporation

**Format for specific study types**<br><br>**Human Subject Research (involving human participants and/or tissue)**<br>• Give the name of the institutional review board or ethics committee that approved the study<br>• Include the approval number and/or a statement indicating approval of this research<br>• Indicate the form of consent obtained (written/oral) or the reason that consent was not obtained (e.g. the data were analyzed anonymously)<br><br>**Animal Research (involving vertebrate animals, embryos or tissues)**<br>• Provide the name of the Institutional Animal Care and Use Committee (IACUC) or other relevant ethics board that reviewed the study protocol, and indicate whether they approved this research or granted a formal waiver of ethical approval<br>• Include an approval number if one was obtained<br>• If the study involved *non-human primates*, add *additional details* about animal welfare and steps taken to ameliorate suffering<br>• If anesthesia, euthanasia, or any kind of animal sacrifice is part of the study, include briefly which substances and/or methods were applied<br><br>**Field Research**<br><br>Include the following details if this study involves the collection of plant, animal, or other materials from a natural setting:<br>• Field permit number<br>• Name of the institution or relevant body that granted permission	
**Data Availability**<br><br>Authors are required to make all data underlying the findings described fully available, without restriction, and from the time of publication. PLOS allows rare exceptions to address legal and ethical concerns. See the PLOS Data Policy and FAQ for detailed information.	Yes - all data are fully available without restriction

A Data Availability Statement describing where the data can be found is required at submission. Your answers to this question constitute the Data Availability Statement and **will be published in the article**, if accepted.<br><br>**Important:** Stating 'data available on request from the author' is not sufficient. If your data are only available upon request, select 'No' for the first question and explain your exceptional situation in the text box.<br><br>Do the authors confirm that all data underlying the findings described in their manuscript are fully available without restriction?	
**Describe where the data may be found in full sentences. If you are copying our sample text, replace any instances of XXX with the appropriate details.**<br><br>• If the data are **held or will be held in a public repository**, include URLs, accession numbers or DOIs. If this information will only be available after acceptance, indicate this by ticking the box below. For example: *All XXX files are available from the XXX database (accession number(s) XXX, XXX.).*<br>• If the data are all contained **within the manuscript and/or Supporting Information files**, enter the following: *All relevant data are within the manuscript and its Supporting Information files.*<br>• If neither of these applies but you are able to provide **details of access elsewhere**, with or without limitations, please do so. For example:<br><br>*Data cannot be shared publicly because of [XXX]. Data are available from the XXX Institutional Data Access / Ethics Committee (contact via XXX) for researchers who meet the criteria for access to confidential data.*<br><br>*The data underlying the results presented in the study are available from (include the name of the third party*	All relevant data are within the manuscript and its Supporting Information files.

and contact information or URL).<br>• This text is appropriate if the data are owned by a third party and authors do not have permission to share the data.<br><br>* typeset	
Additional data availability information:	

Manuscript
Click here to access/download;Manuscript;Health Inequity-PTR 4 COPDs- Conflict Areas.docx

Health Inequity; Possibilities of Initiating Pulmonary Telerehabilitation Programs for Adults with Chronic Obstructive Pulmonary Disorders in Conflict and Low-Resourced Areas; A mixed-methods Phenomenological Study

Suad Ghaben[1,2*], Arimi Fitri Mat Ludin[3&], Badr Elkholi[2,4&], Reem Kullab[4&], Majd Al-Hour[4&], and Devinder Kaur Ajit Singh[1&*]

[1]Physiotherapy Programme & Center for Healthy Ageing & Wellness, Faculty of Health Sciences (H-CARE), Faculty of Health Sciences, Universiti Kebangsaan Malaysia, Jalan Raja Muda Abdul Aziz, 50300 Kuala Lumpur, Malaysia

[2]Department of Physiotherapy, Faculty of Applied Medical Science, Al Azhar University-Gaza, Gaza, Palestine.

[3]Biomedical Science Programme & Center for Healthy Ageing and Wellness (H-CARE), Faculty of Health Sciences, Universiti Kebangsaan Malaysia, Jalan Raja Muda Abdul Aziz, 50300 Kuala Lumpur, Malaysia

[4]Physiotherapy department, Ministry of Health, Gaza, Palestine.

*Corresponding authors:

E-mail: devinder@ukm.edu.my (DKAS)

E-mail: s.ghaben@alazhar.edu.ps (SG)

Abstract:

Background: The "triple problems of COPD"—underdiagnosis, underrecognition, and underdevelopment of pulmonary rehabilitation programs—are global. Pulmonary telerehabilitation (PTR) may solve these problems. This study examines the possibility of launching PTR programs for adults in an area of conflict and low resources.

Methods: A mixed methods convergent design was used to understand outpatient PR unavailability. The medical records of 70 patients with COPD in the MoH chest unit were analysed quantitatively. 13 patients who received in-hospital physiotherapy were questioned via questionnaire, and 12 healthcare professionals (HCPs) were interviewed.

Results: 70 patients with a mean age of 65.3±1.65 years and a mean hospital stay of 4.89±0.392 days were managed with no admission, PT referral, or discharge criteria. Patients reported needing outpatient PR of 4.38 ± 1.193 and a willingness to adopt of 3.77 ±.832. Interviews with HCP highlight the environmental and professional challenges, opportunities, and PTR traits. Quantitative and qualitative data triangulation led to the COPD Sequential Triad (CST) theory.

Conclusion: This study illuminates the national, institutional, and professional PTR development challenges of conflict and low resources, including the sequels of conflict, insufficient resources, and the absence of a structured healthcare system and clinical practice guidelines. Promoting health equity in such areas necessitates that the international community and institutions isolate the healthcare sector from conflicts and apply more effective strategies.

Keywords: Health equity, Pulmonary, telerehabilitation, COPD

Introduction

Chronic Obstructive Pulmonary Disorder (COPD) is the third and sixth leading cause of death (1)] and disability (2)] worldwide, affecting 4% of the population, with higher prevalence and mortality in Lower- and Middle-Income Countries (LMICs) (2, 3). The impact of conflicts on health communities leads to a high incidence of injuries and a lack of disease prevention and health promotion for noncommunicable diseases such as COPD. It also jeopardises the social determinants of health (SDH) and the population's well-being. Conflict leads to shortages in the healthcare system in terms of diminished quality of care, restricted access, and inadequacy of resources and experience (4, 5). Crucially, the conflict has damaged the health infrastructure to an extent that deprived citizens of basic healthcare (6) . Palestine is an occupied territory and a conflict area, ranks third and fourth in the COPD burden in the West Bank and Gaza Strip, accounting for 12.2% and 12.6% of Disability-Adjusted Life Years (DALYs) (7). COPD is the fourth cause of crude mortality in Gaza at 10.4% and the third of years of life lost (YLLs) at 3.1 (7). It had 44,568 COPD cases and 9932 DALYs (8) until 2019. Despite the growing economic and social burden of COPD (1)], it remains a low priority in healthcare policy worldwide (9), particularly in areas of low resources, (10, 11) and affected by conflict and violence (7).

Pulmonary Rehabilitation (PR) in High-Income Countries (HICs) faces multiple challenges, including low referrals, uptake, and adherence [9] and cultural sensitivity affecting PR uptake (12). PR in LMICs are either unavailable (13) or have restricted access (9), restricted stakeholder engagement (11), and unorganised delivery (10). Environmental factors limit access to appropriate programs (14, 15). Personal factors, including health illiteracy [9, 12] and risk factors, impeded PR completion (14, 16, 17). Lack of Health Care Professionals (HCPs) skills and referral standards also hampered PR provision (14). The “triple problems of COPD”: underdiagnosed cases (18), under-recognized disease burden (7, 9-11), and

underdeveloped PR programs (7, 13) Indicate the need for sustainable client-centred PR such as Pulmonary Telerehabilitation (PTR) with technology-based adjuncts. American Thoracic Society (ATS) and European Respiratory Society (ERS) recommended PTR to provide sustainable PR solutions (19). Others claimed that facilitating access to the spirometer detected undiagnosed cases in HICs and LMICs [(2, 18, 20).

PTR is effective in managing COPD symptoms (21). It enhances PR program adherence by increasing accessibility (22, 23) and facilitating deployment with limited resources (24). Remote home monitoring improves treatment outcomes and patient and HCP satisfaction, with HCPs' feedback decreasing hospitalisations (25). TR provides screening, evaluation, supervised intervention, self-management, psychological support, monitoring, and follow-up via mobile or online apps, hardware, and the cloud. Having HCPs actively supervise treatment regimens, assess patient progress, or coordinate care (26). Interactive features or gaming could improve peer support, modelling, and motivation (22). The Smart-OPEP, which was proposed as a form of PTR, integrates the Oscillatory Positive Expiratory Pressure Devices (OPEP) with a spirometer and mobile app and is intended to solve the unavailable PR and enhance OPEP functionalities (27). OPEP improve physiologic and functional outcomes of COPD and may enhance Quality of Life (QoL) (27, 28). Spirometer measurements confirm COPD diagnosis (1) guide emergency department and hospital admission and discharge (29). It also guides the one-month early discharge (30), predicts late post-discharge relapse (31), and strong predictions of mortality and readmission (32)]. Spirometers are still underutilised in COPD diagnosis and management (33), to enhance their utilisation, easy-access portable spirometers were developed (33-36).

Quality care and innovative services require a structured healthcare system (37) with health policy, clinical practice guidelines, health workforce, pharmaceuticals, medical goods, health information systems reporting, governance, and financing (38). The healthcare system in the

occupied territory is fragmented, under-resourced, and technologically outdated; its services are provided by the Ministry of Health (MoH), the United Nations Relief and Works Agency (UNRWA), the private sector, and international Nongovernmental Organizations (INGOs) (39)]. Palestinian healthcare system lacks financing, good governance, evidence-based policies, information and database accessibility and sector coordination (40)]. Current restrictions limit medical supplies, equipment, medications, experts, and patients' movement across the country. During attacks, some healthcare facilities were destroyed and shut down, limiting the MoH's control over land, water, and energy and impeding its ability to provide healthcare (39, 41-43). The lack of outpatient PR in the target MoH prevents patients with COPD from receiving a continuum of care. PTR with technology-based adjuncts may help sustain PR and address healthcare system shortages. This research was conducted to identify [1] challenges, [2] opportunities, and [3] characteristics of initiating PTR programs with technology-based adjuncts.

Material and Methods

Study design

This mixed-methods study integrated quantitative and qualitative research and pragmatism (44, 45), to understand the unavailability of outpatient PR phenomena (46, 47). We collected quantitative data using document analysis and a questionnaire and qualitative data using in-depth interviews (IDI), as shown in Fig 1. This formative research will inform the Smart-OPEP development (27). Using purposive sampling, we adopted the descriptive, cross-sectional design for the quantitative part and the phenomenological method for the qualitative part. A local MoH committee (993123) approved the research protocol. All participants were informed about the study's purpose, data collection process, and telerehabilitation intervention prior. After obtaining informed consent, all questionnaires and IDI were performed through face-to-face individual interviews.

Fig 1: Applying triangulation (convergent parallel MMR) to identify the possibilities of launching PTR.

Data collection

The three study instruments, data extraction table, survey, and interview guides, were prepared after identifying each tool's goal and target population. From 23rd January to 30th January 2022, SG concurrently conducted a preliminary interview with a chest doctor and a physiotherapist to identify their experience with patients diagnosed with COPD and explored randomly selected medical records. Accordingly, SG drafted a version of the study tools, which were refined by the team (SG, BKH, RK, MH, AFML, and DKAS). The questionnaire was validated by three physiotherapy researchers (face and content validity).

Quantitative data

Quantitative data was collected through document analysis and questionnaires.

Document analysis

We analysed medical records of patients with COPD admitted to the MoH chest unit from January 1 until December 31, 2021. Data accession and extraction were performed from 21st February to 9th March 2022 by RK and MH utilising the data extraction table. We acquired permission before accessing medical records. SG reviewed and verified the data from the extraction table. The table encompassed primary data (gender, age, and length of hospital stays) and seven sections, each with a drop-down checklist: [1] admission criteria; [2] Investigations; [3] referral to Physiotherapy (PT); [4] medical management; [5] progress notes; [6] comorbidities; and [7] discharge criteria. The MoH information system allows researchers to access information that identifies individual participants during data collection.

Questionnaire

Data was collected from 7th to 9th March 2022 by RK and MH using the structured and validated questionnaire in an interview context, all participants provided written informed consent. Patients with COPD admitted to the chest unit in 2021, received inpatient physiotherapy and discharged from the hospital were included. The questionnaire has two sections: [1] the demographic data: smoking, comorbidities, medical history, COPD diagnosis date, symptoms, and intervention received post-discharge. [2] The survey included three subsections and measured agreement on a five-point Likert scale, inquiring about [1] inpatient PT management experience, [2] the need for PT hospitalisation, and [3] willingness to receive telerehabilitation. Each subsection was followed by a general inquiry scale, rated 1-5, to asses patients' satisfaction with inpatient PT, the need for PTR, and telerehabilitation willingness. The Arabic survey's alpha Cronbach for the subsections is 0.985, 0.973, and 0.879, indicating high reliability. Content validity (CV) results demonstrate high validity; the I-CVI for the 27 items and the S-CVI scored 1 were calculated using data from three evaluators. The Arabic survey was translated into English for review and verification by AFML and DKAS.

Qualitative data

We collected qualitative data for this study by conducting 12 IDIs from 27th February to 17th March 2022. We interviewed five chest physicians and five physiotherapists who treated patients with COPD. We also interviewed two PT managers who made clinical and administrative decisions. Interviews were conducted in the MoH's participants' offices using interview guides. All three guide versions had three sections: [1] doctors-physiotherapists communication, [2] medical and PT management and [3] PTR need and acceptance. SG obtained the informed consent and moderated the interviews, and BKH took notes and audio-recorded them. BKH prearranged all interviews via phone and sent participants information sheets.

Data analysis

Quantitative data

Medical records and questionnaires were analysed using IBM SPSS 22. Records about participant age, gender, hospital stay, medical investigation, management, comorbidities, and discharge criteria were analysed using descriptive statistics. Inferential statistics were deployed to analyse the correlation between referral to PT and dyspnea, coughing, and sputum production. Questionnaire data identified participants' gender, age, length of COPD diagnosis, comorbidities, last week's COPD symptoms, and hospital discharge care using descriptive statistics.

Qualitative data

Findings were reported according to the Consolidated Criteria for Reporting Qualitative Research (COREQ) (48). MH and RK transcribed verbatim the Arabic recorded interviews, then denaturistically translated them into English (49). SG and BKH, line by line, reviewed and verified the interview scripts. SG used the NVivo 12 to categorise data into initial codes, merge and shift codes into categories, and produce themes from categories to answer the RQ (50, 51). Codes and themes were phrases or full sentences that captured the essence of the extracts. SG discussed themes with DKAS, AFML, and BKH until an agreement was reached. Member checking and triangulation verified thematic analysis (52, 53); participants’ trust and rapport were attained through prolonged engagement; researchers and HCPs were involved for years in variable projects (53).

Triangulation

A well-justified MMR design and suitable triangulation techniques are essential for credible and rigorous results (54, 55). This study used triangulation and reporting recommendations

(54, 56-58), to organise and integrate the qualitative and quantitative data, enrich the data, and verify the results.

Results

The study results were derived from document analysis, questionnaires, and IDI. These results were triangulated in the Convergent parallel MMR triangulation model.

Document analysis

Participants

Document analysis and demographics of the questionnaire are shown in Table 1 in S1 Tables.

The study comprised 70 medical records of patients with COPD hospitalised in the chest unit. For two females and 68 males, their average age and hospital stay were 65.3±1.65 years and 4.89±0.392 days, respectively.

The top five criteria for admitting patients to the chest unit were SPO2, dyspnea, coughing, respiratory rate and high temperature, recorded in 90%, 75.7%, 71.4%, 51.4%, and 42.8% of the records, respectively. In 91.4%, 75.7%, 72.8%, 72.8%, and 71.4% of records, CBC, ECG, electrolytes, ABGs, and chest X-ray were the top five investigation procedures. Spirometry and bronchoscope were never used for investigation. Although admission and investigation outcomes were consistent, they didn't follow any guidelines. The lack of consistently validated symptom scales for COPD diagnosis and GOLD guidelines is evident. The most commonly prescribed medications were supplements, antibiotics, inhaled bronchodilators, O2 therapy, inhaled antispasmodics and antibiotics in 84.3%, 78.6%, 57.1%, 47.1%, 40%, and 32.8%. PT was inadequately represented in patients' records, with just 7.1% showing a written referral and 10% recording a verbal “discussion with physiotherapist”. In contrast, 82.8% of medical records did not mention PT.

Patients usually have COPD and other comorbidities. Hypertension, congestive heart failure, type-2 diabetes, and smoking were the most prevalent comorbidities in 45.7%, 32.9%, 31.4%, and 28.6% of medical records. In 51.4%, 30%, and 27.1% of the records, discharge decisions were based on increased SPO2, dyspnea alleviation, and chest clearance, respectively. Improved body temperature, heart rate, and respiration rate were recorded in 30%, 24.3%, and 20% of records. Only 21.4% and 1.4% of records listed air entry and lung function tests, the most critical indications of improved COPD.

The chi-square test examined the association between PT referral, the COPD triad, dyspnea, cough, and secretion production. 68.6% of patients in the chest unit were not referred to PT. 62.3%, 64%, and 70% (n=70) of dyspnea, coughing, and sputum patients were not referred to PT. The association between dyspnea and the likelihood that patients are being referred to PT was significant ($p < 0.05$), while it was insignificant ($p > 0.05$) for coughing and sputum production.

Questionnaire

Participants

13 patients with COPD admitted to the chest unit and received in-hospital PT completed the questionnaire. The average age was 66,07±9.84 years, BMI was 35.2±13.35, and years since diagnosis with COPD were 6.88±7,52 years. Most patients had low-level education, while 7.7% had a university degree. 7.7% were employed.

Walking difficulties, stress and anxiety, diabetes, hypertension, cardiovascular disorders, and current smoking were reported by 76.9%, 61.5%, 53.8%, 46.2%, and 38.5% of respondents. Dyspnea, fatigue, inability to conduct ADL, coughing, and sputum were reported by 92.3%, 84.6%, 84.6%, 69.2%, and 69.2% of participants, respectively. About 23.1% of participants

continued to receive PT after discharge, mostly private sessions. In comparison, 100% used drugs to treat COPD, and 46.2% consulted a chest doctor.

Patients' in-hospital PT experience is illustrated in Table 2 in S1 Tables, outpatient PR needs, and PTR adoption. In-hospital PT patients had moderate acceptance of care quality, with mean values ranging from 2.31 ± 1.377 to 2.69 ± 1.653 for specific services. They reported lower levels of satisfaction with care (2.15 ± 1.573). Patients valued post-discharge PT, with mean values ranging from 3.77 ± 1.481 to 4.54 ± 1.19, and overall need scored a higher value of 4.38 ± 1.193. Patients were willing to adopt telerehabilitation services, with mean values ranging from 3.23 ± 1.013 to 4.31 ± 1.032 for specific tools and an overall willingness of 3.77 ±.832. Patients who preferred home PT, either conventional or TR, scored highest, with mean values of 4.54 ± 1.198 and 4.31 ± 1.032, respectively.

The In-depth interviews

Participants

Five chest physicians (average experience: 20±7.03 years) and five physiotherapists (average: 11±.7.74 years) were interviewed. The PT department director and the PT clinics' general directorate at MoH were also interviewed.

Three categories of possibilities for launching PTR in the target area were identified: challenges, opportunities, and characteristics of PTR. Fig 2 shows the thematic framework; coded interview transcripts identified five themes: environmental challenges, professional challenges, environmental opportunities, and characteristics of PTR. Each theme was described into subthemes, with a total of 25 subthemes.

Fig 2: Thematic framework. (-) indicates lack of. PT: Physiotherapy, PR: Pulmonary Rehabilitation, PTR: pulmonary telerehabilitation

Challenges were classified into environmental and professional challenges, with participants' numbers and sample quotations for each subtheme shown Table 3 in S1 Tables. Physiotherapists were referred to P1 to P5, doctors to D1 to D5, and PT managers to M1 and M2. Five subthemes described the environmental challenges; “Telerehabilitation is not a priority in the MoH practice”, “Physiotherapy is under-prioritised in MoH plans and practice”, “The impacts of conflict lead to unavailability of resources”, “The unavailability of resources influenced the COPD management”, and “The heavy burden of COPD management is exacerbated by emergencies. “Seven subthemes described the professional challenge; “Work overload for doctors & physiotherapists”, “Doctors underestimate the roles of PT”, “lack of referral criteria to PT”, “Lack of professionals' efforts to enhance experience and collaboration”, “Lack of shared discharge decision”, “Improper PT documentation”, and “Difficulties in modifying patients’ risk factors”.

Opportunities were classified into environmental and professional, as shown in Table 4 in S1 Tables. Three subthemes described the environmental opportunity; “Possibilities to adapt health policies in MoH to provide PR and PTR services”, “MoH values the significance of PR and PTR”, and “Managers in MoH support professional development and collaboration.” five subthemes described the professional opportunities; “The perceived need for the post-discharge PR”, “Why PTR is essential for the management of COPD?”, “Possibilities to enhance professional practice”, “possibilities to enhance interprofessional collaboration”, and “Willingness to adopt PTR among HCPs”.

Five subthemes described characteristics of the intended PTR, as shown in Table 5 in S1 Tables; “Therapeutic devices are recommended “, Mobile app is a potential tool for PTR”, “Visual and audio contents are preferred”, “Requirements to launch the PTR”, and “Hybrid PTR regime is preferred”.

The triangulation model

This convergent parallel study included results from records, questionnaires, and interviews to investigate the challenges and opportunities of launching PTR. The integrated results added to the literature on the "triple problems of COPD": underdiagnosis (18), disease under-recognition (7, 9-11), and PTR underdevelopment (7, 13). The HCPs highlighted the underdiagnosis: "almost two-thirds are COPD but not diagnosed." Fig 3 depicts the disease under-recognition and PR underdevelopment according to the COPD Sequential Triad (CST).

Fig 3: The triangulation model represents the CST model based on the present study's findings. Three key factors primarily impact the triple problems associated with COPD. The conflict area, the low-resource setting, and the socioeconomic factors influencing the population serve as illustrations at the national level. (2) At the institutional level, there is a lack of a structured healthcare system, medical dominance, and policies that undervalue PR. The absence of clinical practice guidelines and professional regulations and the overloading issue contribute to the professional factor. (-) indicates lack of HCS: healthcare system

At the national level, three factors influenced the triple COPD challenges: the conflict and its sequels, as stated by four HCPs: "the siege"; the low-resourced settings: "the unavailability of resources"; and the socioeconomic influence: "the heavy burden of COPD is exacerbated by emergencies" and "difficulties in modifying patients' risk factors."

Document analysis revealed a lack of institutional clinical practice guidelines and standardised admission criteria. Nine HCPs reported a "lack of referral criteria to PT," and document analysis supported that. Ten HCPs reported a "lack of shared discharge decision" "once the patient improved." Unconsolidated discharge criteria were supported by document analysis. The highest score was 50.4% for SPO2 in patient records. Although discharge planning was not mentioned in patients' records, the questionnaire showed that all discharged

patients continued their medications, 46.2% continued physician follow-ups, and 23.1% continued PT. HCPs indicated, “I created a follow-up program with some patients” and “simply referred patients to the outpatient clinic.”

The quality of in-hospital PT indicated a "lack of professionals' efforts to enhance experience and collaboration" by five HCPs, while patient satisfaction was poor (2.15 ± 1.573). Eight HCPs recorded “The Perceived Need for Post-Discharge PR” for outpatient PR, and three recorded “The MoH, staff values the significance of PR and PTR”. Patients emphasised this (4.38 ± 1.193). Nine HCPs recorded the importance of PTR in COPD management, and patients indicated a willingness to adopt it (mean value of 3.77 ±.832). Additionally, eleven individuals indicated a “willingness to adopt PTR among HCPs.”

Nine HCPs provided PTR characteristics, indicating willingness to use the mobile app and patient support (mean value of 3.23 ± 1.301). Five HCPs and patients reported using OPEP as an additional treatment device (mean value: 3.69 ± 1.182). Two HCPs chose the PTR program's visual and auditory components, and patients preferred learning therapeutic exercise through video (mean value = 3.77 ± 1.013).

Discussion

This study highlighted the environmental and professional challenges, opportunities, and characteristics of initiating PTR in a conflict and low-resource area. It also outlined the national, institutional, and professional factors that influence the initiation of PTR.

Our analysis stated that the target area's conflict status and low resources hinder PTR implementation, impacting the country's socioeconomic status. Thus, resource constraints led the MoH to eliminate TR from current and future practice, which aligns with previous findings. [(43, 59) and alarms about national, institutional, and humanitarian crises due to the impacts of conflict (6, 41), leading to the devastation of the MoH (6, 43, 59-61). Due to the

impacts of conflict, and uneven distribution of power, money, and resources among the healthcare system's pillars, led to lowering the QOL (59, 61, 62)], which has greatly affected social determinants of health and health outcomes (63, 64). The WHO recommends improving daily living, addressing power, money, and resource inequalities, and monitoring and evaluating the problem and required action to improve health equity and outcomes (63). The latter seems most feasible for researchers, while affecting daily life and power, money, and service inequalities are beyond this discussion, especially given discouraging past experiences[(65)].

At the institutional level, the MoH's possibility of partnering with UNRWA and INGOs to provide PR. Despite specific issues, these institutions clearly shape this healthcare system. UNRWA (5), and the International Committee of the Red Cross (ICRC) (4), have focused on providing the occupied territory with vital healthcare, offering flexibility, richness, and variability of services and expertise (4, 5, 66). The MoH capitalise on these institutions and their assets by maintaining sovereignty, self-determination, and justice, a power mark disputed by these parties (59-61). This complex equation can only be tackled if the MoH's leaders exercise power wisely, develop logical plans, and contain national expertise rather than embrace a factional policy. A call to sign a code of conduct that emphasises the MoH's policies and plans might also be considered.

At the professional level, we captured the non-adoption of clinical practice guidelines; HCPs did not disclose their adoption, and guidelines were not documented across the patients' records, which might indicate a documentation system defect or non-adoption of guidelines. Here, we argue that doctors and physiotherapists employ their experience in an ad hoc manner to treat their patients. Therefore, it's crucial to adopt and adhere to a clinical practice guideline (67)], as noncompliance would impact care quality (68-70). Documentation in PT is crucial to improving patient care, providing proof of business and legal records and evidence of

practice, enhancing HCPs' communication, guiding clinical decision-making (71), and improving quality through records audits (72). However, documentation is not preferred by physiotherapists due to a lack of time, skills, and rewards (71, 73, 74), resulting in inconsistent and heading-only records (75, 76). To address these concerns, the Federated Electronic Health Records (FEHR) should be established (77), which is a nationwide system that encompasses electronic health records of variable disciplines and functionalities, gathering all sectors together to enhance efficiency, leverage telehealth and help establish PTR (77). Considering the professional issues, we highlighted the high awareness of HCPs and patients about the existing need for PR and PTR and their willingness to adopt PTR. Awareness and willingness are crucial in the technology acceptance and adoption process (78, 79), and should be invested in building the human resources to create the PTR programs.

The “physician physiotherapy” professional dilemma is prominent in this study. We highlighted the “physiotherapy is under-prioritised in MoH plans and practice”, “Doctors underestimate the role of PT”, “lack of professionals' efforts to enhance the experience and interprofessional collaboration”, “lack of referral criteria to PT”, “the lack of shared discharge decision”; and “improper PT documentation”. An intensive analysis of these themes indicated that the medical role influences COPD management more than PT. Although doctors recognised PT, other themes indicated that this is not the actual practice. This contradiction might be attributed to the good performance of physiotherapists during the COVID-19 emergency. Spotlighting the “physician physiotherapy” professional dilemma, PT has moved from secondary healthcare to an autonomous practitioner, known as direct access, allowing physiotherapists to treat patients without a referral, which was authorised by The World Physiotherapy (WCPT) in 2011 (80); however, the direct access system has been challenged by several factors mainly medical dominance (81). Notably, 58% of WCPT member organisations had direct access until 2012, especially in the private sector, with policymakers'

and physicians' restrictions (82)], and several countries still consider direct access (83-85). Direct access is cost-effective (86)], owing to its benefits of reducing time, cost, and resources (85) and improving disability and QoL (87). In the current MoH practice, doctors tell physiotherapists what and how to treat their patients: “say cough,” Physiotherapists complain of being marginalised by both the MoH and doctors; “doctors will not provide space for physiotherapists to work.” Regular referral-based PT has limited physiotherapists’ clinical reasoning, decision-making, and evidence-based practice, resulting in considerable irresponsibility and frustration (81)], which was revealed in “physiotherapists’ limited efforts to enhance their experience or collaboration with doctors.” The development of the PT profession and direct access has been challenged by a lack of professional regulations, medical dominance in the hierarchical structure, and inadequate education standards to meet competency requirements (81). Efforts to upscale professional development and autonomy in the current MoH practice have always been challenged by the MoH’s medical hierarchy and the lack of professional regulations and clinical practice guidelines. Direct access is possible, and doctors-physiotherapists partnering could build a suitable paradigm (88). Professional regulation and clinical practice guidelines are crucial for interprofessional collaboration, professional boundaries, evidence-based practice and sound professional judgment.

The CST illustrates the national, institutional, and professional challenges to PTR development in a conflict and low-resourced area. Some challenges are common with other LMICs (89, 90), others are exclusive, making the CST crucial. The CST would help policymakers, researchers, and HCPs bridge healthcare shortages; however, political and socioeconomic factors are beyond their control. Considering the CST and the WHO Commission on the SDH to promote health equity (63). We argue that limiting the action to “evaluate the problem and the required action” would never be satisfactory for promoting health equity and launching the PTR in the target area. Therefore, the international

community must isolate the healthcare sector from political conflicts, call on international institutions to work for healthcare equity, and, above all, ensure the warring parties uphold their obligations under international humanitarian law (6).

This study explored the possibility of launching PTR in an area of conflict and low resources, considering these exclusive settings, particularly the impacts of conflict. We argue that the inconsistent healthcare quality globally would make it harder to launch TR in LMICs (91), whereas HICs prioritise high technologies, data privacy, safety, usability, feasibility, affordability, and patient preferences (92), LMICs prioritise TR infrastructures (93) and health and digital health literacy (91). In the current MoH policies and practice, the conflict and violence worsened the situation, making it crucial to question whether the right to "consistent quality of healthcare worldwide" would leverage building PTR (63, 94, 95) or whether people in conflict areas who struggle to get medications would be criticised for benefiting from a demanding PTR.

Future research

This work lacks the PTR program specifications. It would be advisable to develop, evaluate, and compare the acceptance, engagement, and effectiveness of variable PTR programs in the target areas. And explore other conflict areas for further analysis.

Strengths of the study

This study analyses the global and national shortages of the COPD management phenomena. We expect policymakers, researchers, and HCPs to use this work to reform the healthcare system, fill the shortages, build plans, initiate PTR and provide the best possible quality of care.

Limitations of the study

The IDI was conducted in Arabic to allow open discussion. None of the data analysis software could handle Arabic transcripts, so we had to translate and verify them into English, which consumed time and effort and complicated the qualitative data analysis.

Conclusion

This study addressed the possibility of launching PTR in areas of conflict and low resources, like the occupied territory. The primary issues preventing service needs and TR's establishment are the nonpriority in MoH practice and the fragmented COPD patient management. The MoH might cooperate with NGOs and alter its policies to provide PTR services. Additionally, interprofessional collaboration may be needed to implement PTR. Supporting health equity necessitates the international community and INGOs to put aside the healthcare sector from conflicts and apply more efficient strategies.

Acknowledgements

The authors would like to acknowledge all study participants for their contributions. We thank the managers at the MoH for enabling the organisation of IDIs and all colleagues for facilitating the discussions and data collection.

Authors' contribution

SG conducted a preliminary interview with a chest doctor and a physiotherapist. SG prepared the draft version of the three study tools accordingly. SG and BKH conducted the IDI with HCPs; SG moderated the interviews, and BKH took notes and audio-recorded the meetings. RK and MH performed data extraction using the data extraction table, which SG reviewed and verified. RK and MH collected questionnaire data from patients in an interview context. The interview audios with HCPs were transcribed verbatim by MH and RK and then translated into English by MH. SG and BKH revised and verified the interview scripts. SG generated codes. SG drafted the manuscript. The research team SG, BKH, RK, MH, AFML, and DKAS discussed and verified the study tools, initial codes, and themes, and revised and verified the manuscript.

References:

1. GOLD. Global Initiative for Chronic Obstructive Pulmonary Disease. USA; 2023.
2. Forum of International Respiratory Societies. The Global Impact of Respiratory Disease. . 2021.
3. World Health Organization. Chronic obstructive pulmonary disease (COPD) 2022 [Available from: https://www.who.int/news-room/fact-sheets/detail/chronic-obstructive-pulmonary-disease-(copd).
4. Barth CA, Wladis A, Blake C, Bhandarkar P, O'Sullivan C. Users of rehabilitation services in 14 countries and territories affected by conflict, 1988–2018. Bulletin of the World Health Organization. 2020;98(9):599.
5. Kitamura A, Jimba M, McCahey J, Paolucci G, Shah S, Hababeh M, et al. Health and dignity of Palestine refugees at stake: a need for international response to sustain crucial life services at UNRWA. The Lancet. 2018;392(10165):2736-44.
6. Asi Y, Mills D, Greenough PG, Kunichoff D, Khan S, Hoek JVD, et al. 'Nowhere and no one is safe': spatial analysis of damage to critical civilian infrastructure in the Gaza Strip during the first phase of the Israeli military campaign, 7 October to 22 November 2023. Conflict and Health. 2024;18(1):24.
7. Mosleh M, Aljeesh Y, Dalal K. Burden of chronic diseases in the Palestinian healthcare sector using disability-adjusted life years (DALY), Palestine. Diversity and equality in health and care. 2016;13(3):261-8.
8. Feizi H, Alizadeh M, Nejadghaderi SA, Noori M, Sullman MJ, Ahmadian Heris J, et al. The burden of chronic obstructive pulmonary disease and its attributable risk factors in the Middle East and North Africa region, 1990–2019. Respiratory Research. 2022;23(1):1-15.

9. Halpin DM, Celli BR, Criner GJ, Frith P, Varela MVL, Salvi S, et al. It is time for the world to take COPD seriously: a statement from the GOLD board of directors. Eur Respiratory Soc; 2019.
10. Orme MW, Free RC, Manise A, Jones AV, Akylbekov A, Barton A, et al. Global RECHARGE: Establishing a standard international data set for pulmonary rehabilitation in low-and middle-income countries. Journal of global health. 2020;10(2).
11. Habib GM, Uzzaman MN, Malik P, Rabinovich R, Uddin A, Alam SR, et al. Engaging with stakeholders in a research programme to promote implementation of pulmonary rehabilitation in Bangladesh: Challenges and opportunities. Journal of Global Health. 2020;10(2).
12. Levack WM, Jones B, Grainger R, Boland P, Brown M, Ingham TR. Whakawhanaungatanga: the importance of culturally meaningful connections to improve uptake of pulmonary rehabilitation by Māori with COPD–a qualitative study. International journal of chronic obstructive pulmonary disease. 2016:489-501.
13. Singh SJ, Halpin DM, Salvi S, Kirenga BJ, Mortimer K. Exercise and pulmonary rehabilitation for people with chronic lung disease in LMICs: challenges and opportunities. The Lancet Respiratory Medicine. 2019;7(12):1002-4.
14. Lahham A, Holland AE. The need for expanding pulmonary rehabilitation services. Life. 2021;11(11):1236.
15. Cox NS, Oliveira CC, Lahham A, Holland AE. Pulmonary rehabilitation referral and participation are commonly influenced by environment, knowledge, and beliefs about consequences: a systematic review using the Theoretical Domains Framework. Journal of physiotherapy. 2017;63(2):84-93.
16. Keating A, Lee A, Holland AE. What prevents people with chronic obstructive pulmonary disease from attending pulmonary rehabilitation? A systematic review. Chronic respiratory disease. 2011;8(2):89-99.
17. Gaveikaite V, Grundstrom C, Winter S, Schonenberg H, Isomursu M, Chouvarda I, et al. Challenges and opportunities for telehealth in the management of chronic obstructive pulmonary disease: a qualitative case study in Greece. BMC Medical Informatics and Decision Making. 2020;20(1):1-12.
18. Carlone S, Balbi B, Bezzi M, Brunori M, Calabro S, Foschino Barbaro MP, et al. Health and social impacts of COPD and the problem of under-diagnosis. Multidisciplinary respiratory medicine. 2014;9(1):1-6.
19. Holland AE, Cox NS, Houchen-Wolloff L, Rochester CL, Garvey C, ZuWallack R, et al. Defining modern pulmonary rehabilitation. An official American Thoracic Society workshop report. Annals of the American Thoracic Society. 2021;18(5):e12-e29.
20. Labaki WW, Han MK. Chronic respiratory diseases: a global view. The Lancet Respiratory Medicine. 2020;8(6):531-3.
21. Cox NS, Dal Corso S, Hansen H, McDonald CF, Hill CJ, Zanaboni P, et al. Telerehabilitation for chronic respiratory disease. Cochrane Database of Systematic Reviews. 2021(1).
22. Hoaas H, Andreassen HK, Lien LA, Hjalmarsen A, Zanaboni P. Adherence and factors affecting satisfaction in long-term telerehabilitation for patients with chronic obstructive pulmonary disease: a mixed methods study. BMC medical informatics and decision making. 2016;16(1):1-14.
23. Hansen H, Bieler T, Beyer N, Kallemose T, Wilcke JT, Østergaard LM, et al. Supervised pulmonary tele-rehabilitation versus pulmonary rehabilitation in severe COPD: a randomised multicentre trial. Thorax. 2020;75(5):413-21.
24. Jácome C, Marques A, Oliveira A, Rodrigues L, Sanches I. Pulmonary telerehabilitation: An international call for action. Pulmonology. 2020;26(6):335.
25. Nagase FI, Stafinski T, Avdagovska M, Stickland MK, Etruw EM, Menon D. Effectiveness of remote home monitoring for patients with Chronic Obstructive Pulmonary Disease (COPD): systematic review. BMC Health Services Research. 2022;22(1):1-25.
26. Ghaben SJ, Mat Ludin AF, Mohamad Ali N, Beng Gan K, Singh DKA. A framework for design and usability testing of telerehabilitation system for adults with chronic diseases: A panoramic scoping review. DIGITAL HEALTH. 2023;9:20552076231191014.

27. Ghaben SJ, Aqel MO, Sama'a AB, Sabha WHA, Hammad RKA, Al Zaq ZM, et al., editors. Aerobika as an Evidence Based Physiotherapy Procedure for COPD and the Proposition of a Smart OPEP Device. 2020 International Conference on Assistive and Rehabilitation Technologies (iCareTech); 2020: IEEE.
28. Sahardin SN, Jailaini MFM, Abeed NNN, Ban AY-L, Hau NB, Azmel AA, et al. Impact of Aerobika® oscillating positive expiratory pressure in improving small airway resistance, lung function, symptoms and exercise capacity in chronic obstructive pulmonary disease. Frontiers in Medicine. 2023;10:1113.
29. Emerman CL, Effron D, Lukens TW. Spirometric criteria for hospital admission of patients with acute exacerbation of COPD. Chest. 1991;99(3):595-9.
30. Rea H, Kenealy T, Adair J, Robinson E, Sheridan N. Spirometry for patients in hospital and one month after admission with an acute exacerbation of COPD. International Journal of Chronic Obstructive Pulmonary Disease. 2011:527-32.
31. Loh CH, Genese FA, Kannan KK, Lovings TM, Peters SP, Ohar JA. Spirometry in hospitalized patients with acute exacerbation of COPD accurately predicts post discharge airflow obstruction. Chronic Obstructive Pulmonary Diseases: Journal of the COPD Foundation. 2018;5(2):124.
32. Flattet Y, Garin N, Serratrice J, Perrier A, Stirnemann J, Carballo S. Determining prognosis in acute exacerbation of COPD. International journal of chronic obstructive pulmonary disease. 2017:467-75.
33. Exarchos KP, Gogali A, Sioutkou A, Chronis C, Peristeri S, Kostikas K. Validation of the portable Bluetooth® Air Next spirometer in patients with different respiratory diseases. Respiratory research. 2020;21:1-7.
34. Barr RG, Stemple KJ, Mesia-Vela S, Basner RC, Derk SJ, Henneberger PK, et al. Reproducibility and validity of a handheld spirometer. Respiratory care. 2008;53(4):433-41.
35. Ramos Hernández C, Núñez Fernández M, Pallares Sanmartín A, Mouronte Roibas C, Cerdeira Domínguez L, Botana Rial MI, et al. Validation of the portable air-smart spirometer. PloS one. 2018;13(2):e0192789.
36. Represas-Represas C, Fernández-Villar A, Ruano-Raviña A, Priegue-Carrera A, Botana-Rial M, settings" sgoVoC-in-sh. Screening for chronic obstructive pulmonary disease: validity and reliability of a portable device in non-specialized healthcare settings. PLoS One. 2016;11(1):e0145571.
37. World Health Organization. Everybody business : strengthening health systems to improve health outcomes : WHO's framework

for action. World Health Organization, 20 Avenue Appia, 1211 Geneva 27, Switzerland; 2007. Contract No.: ISBN 978 92 4 159607 7.
38. World Health Organization. MONITORING THE BUILDING BLOCKS OF HEALTH SYSTEMS: A HANDBOOK OF INDICATORS AND THEIR MEASUREMENT STRATEGIES. World Health Organization, 20 Avenue Appia, 1211 Geneva 27, Switzerland; 2010. Contract No.: ISBN 978 92 4 156405 2.
39. AlKhaldi M, Kaloti R, Shella D, Al Basuoni A, Meghari H. Health system's response to the COVID-19 pandemic in conflict settings: Policy reflections from Palestine. Global public health. 2020;15(8):1244-56.
40. AlKhaldi M, Alkaiyat A, Abed Y, Pfeiffer C, Halaseh R, Salah R, et al. The Palestinian health research system: who orchestrates the system, how and based on what? A qualitative assessment. Health research policy and systems. 2018;16(1):1-15.
41. World Health Organization. Health conditions in the occupied Palestinian territory, including east Jerusalem, and in the occupied Syrian Golan. WHO; 2023 17, May. Contract No.: A76/15.
42. United Nations Office for the Coordination of Humanitarian Affairs. Humanitarian situation in Gaza | Flash Update #5 as of 17:00, 15 May 2023: OCHA; 2023 [cited 2023 3rd, August]. Available from: https://www.ochaopt.org/content/flash-update-5-15-may-2023.
43. Mosleh M, Aljeesh Y, Dalal K, Eriksson C, Carlerby H, Viitasara E. Perceptions of non-communicable disease and war injury management in the Palestinian health system: a qualitative study of healthcare providers perspectives. Journal of Multidisciplinary Healthcare. 2020:593-605.

44. Creswell JW, Poth CN. Philosophical Assumptions and Interpretive Frameworks. In: Helen Salmon JS, Chelsea Pearson, Libby larson, editor. Qualitative inquiry and research design: Choosing among five approaches

Fourth ed. United States of America: Sage publications; 2018. p. 15-38.
45. Creswell JW, Creswell JD. Mixed Methods Procedures. In: Chelsea Neve MOH, David C. Felts, Amy Marks., editor. Research design: Qualitative, quantitative, and mixed methods approaches. Fifth ed. United States of America: Sage publications; 2018. p. 294-327.
46. Martiny KM, Toro J, Høffding S. Framing a phenomenological mixed method: from inspiration to guidance. Frontiers in Psychology. 2021;12:602081.
47. Creswell JW, Poth CN. Phenomenological Research. In: Helen Salmon JS, Chelsea Pearson, Libby larson, editor. Qualitative inquiry and research design: Choosing among five approaches. Fourth ed. United States of America: Sage publications; 2018. p. 75-80.
48. Tong A, Sainsbury P, Craig J. Consolidated criteria for reporting qualitative research (COREQ): a 32-item checklist for interviews and focus groups. International journal for quality in health care. 2007;19(6):349-57.
49. Widodo HP. Methodological considerations in interview data transcription. International Journal of Innovation in English Language Teaching and Research. 2014;3(1):101-7.
50. Castleberry A, Nolen A. Thematic analysis of qualitative research data: Is it as easy as it sounds? Currents in pharmacy teaching and learning. 2018;10(6):807-15.
51. Saunders CH, Sierpe A, von Plessen C, Kennedy AM, Leviton LC, Bernstein SL, et al. Practical thematic analysis: a guide for multidisciplinary health services research teams engaging in qualitative analysis. BMJ. 2023;381:e074256.
52. Jonsen K, Jehn KA. Using triangulation to validate themes in qualitative studies. Qualitative research in organizations and management: an international journal. 2009;4(2):123-50.
53. Hadi MA, José Closs S. Ensuring rigour and trustworthiness of qualitative research in clinical pharmacy. International journal of clinical pharmacy. 2016;38:641-6.
54. Turner SF, Cardinal LB, Burton RM. Research design for mixed methods: A triangulation-based framework and roadmap. Organizational Research Methods. 2017;20(2):243-67.
55. Dupin CM, Borglin G. Usability and application of a data integration technique (following the thread) for multi-and mixed methods research: A systematic review. International Journal of Nursing Studies. 2020;108:103608.
56. Creswell JW, Tashakkori A. Developing publishable mixed methods manuscripts. Sage Publications Sage CA: Los Angeles, CA; 2007. p. 107-11.
57. Fetters MD, Curry LA, Creswell JW. Achieving integration in mixed methods designs—principles and practices. Health services research. 2013;48(6pt2):2134-56.
58. Steinmetz-Wood M, Pluye P, Ross NA. The planning and reporting of mixed methods studies on the built environment and health. Preventive medicine. 2019;126:105752.
59. Smith RJ. Healthcare under siege: Geopolitics of medical service provision in the Gaza Strip. Social Science & Medicine. 2015;146:332-40.
60. Giacaman R, Khatib R, Shabaneh L, Ramlawi A, Sabri B, Sabatinelli G, et al. Health status and health services in the occupied Palestinian territory. The lancet. 2009;373(9666):837-49.
61. Becker A, Al Ju'beh K, Watt G. Keys to health: justice, sovereignty, and self-determination. The Lancet. 2009;373(9668):985-7.
62. Rosenthal FS. A comparison of health indicators and social determinants of health between Israel and the Occupied Palestinian Territories. Global Public Health. 2021;16(3):431-47.
63. World Health Organization. Commission on Social Determinants of Health FINAL REPORT | EXECUTIVE SUMMARY. 20 Avenue Appia, 1211 Geneva 27, Switzerland WHO; 2008. Report No.: WHO/IER/CSDH/08.1.
64. Remington PL, Catlin BB, Gennuso KP. The county health rankings: rationale and methods. Population health metrics. 2015;13(1):1-12.
65. Rasanathan K. 10 years after the Commission on Social Determinants of Health: social injustice is still killing on a grand scale. The Lancet. 2018;392(10154):1176-7.

66. Keelan E. Medical care in Palestine: working in a conflict zone. The Ulster medical journal. 2016;85(1):3.
67. Surani S, Aiyer A, Eikermann S, Murphy T, Anand P, Varon J, et al. Adoption and adherence to chronic obstructive pulmonary disease GOLD guidelines in a primary care setting. SAGE Open Medicine. 2019;7:2050312119842221.
68. Maniscalco M, Martucci M, Fuschillo S, de Felice A, D'Anna SE, Cazzola M. A case scenario study on adherence to COPD GOLD recommendations by general practitioners in a rural area of southern Italy: the "progetto PADRE". Respiratory Medicine. 2020;170:105985.
69. Desalu OO, Onyedum CC, Adeoti AO, Gundiri LB, Fadare JO, Adekeye KA, et al. Guideline-based COPD management in a resource-limited setting—physicians' understanding, adherence and barriers: a cross-sectional survey of internal and family medicine hospital-based physicians in Nigeria. Primary Care Respiratory Journal. 2013;22(1):79-85.
70. Calle Rubio M, Alcázar Navarrete B, Soriano JB, Soler-Cataluña JJ, Rodriguez Gonzalez-Moro JM, Fuentes Ferrer ME, et al. Clinical audit of COPD in outpatient respiratory clinics in Spain: the EPOCONSUL study. International Journal of Chronic Obstructive Pulmonary Disease. 2017:417-26.
71. Quinn L, Gordon J. Documentation for rehabilitation-E-book: A guide to clinical decision making in physical therapy: Elsevier Health Sciences; 2015.
72. Sparkes V. Treatment of low back pain: monitoring clinical practice through audit. Physiotherapy. 2005;91(3):171-7.
73. Buyl R, Nyssen M. Structured electronic physiotherapy records. International journal of medical informatics. 2009;78(7):473-81.
74. Richoz C, Ayer A, Berchtold A, Richoz S. Record keeping by Swiss physiotherapists: A national survey of knowledge regarding legal requirements. Swiss medical weekly. 2011.
75. Partia R. The content and structure of electronic health records in physiotherapy. Physiotherapy. 2015;101:e1179-e80.
76. Häyrinen K. Evaluation of Physiotherapists' Documentation: a Retrospective Record Analysis before the Implementation of National Recommendations in the EHR system. Electronic Journal of Health Informatics. 2014;8(1).
77. El Jabari C, Macedo M, Al-Jabari MO, editors. Towards a new paradigm of federated electronic health records in Palestine. Informatics; 2020: MDPI.
78. Frambach RT, Schillewaert N. Organizational innovation adoption: A multi-level framework of determinants and opportunities for future research. Journal of business research. 2002;55(2):163-76.
79. Niknejad N, Ismail W, Bahari M, Nazari B. Understanding telerehabilitation technology to evaluate stakeholders' adoption of telerehabilitation services: A systematic literature review and directions for further research. Archives of Physical Medicine and Rehabilitation. 2021;102(7):1390-403.
80. World Physiotherapy. Direct access and patient/client self-referral to physical therapy. Policy statement. 2019.
81. Lim WS, Sharma S, Devan H. Physiotherapists' attitudes towards and challenges of working in a referral-based practice setting–a systematic scoping review. European Journal of Physiotherapy. 2021;23(6):332-43.
82. Bury TJ, Stokes EK. A global view of direct access and patient self-referral to physical therapy: implications for the profession. Physical therapy. 2013;93(4):449-59.
83. Alosaimi SM, Alamri RA, Alghamdi MA. Attitudes and Beliefs of Physical Therapists in Saudi Arabia Regarding Direct Access and Scope of Practice. Integrative Journal of Medical Sciences. 2022;9:1-6.
84. Alnaqbi A, Shousha T, AlKetbi H, Hegazy FA. Physiotherapists' perspectives on barriers to implementation of direct access of physiotherapy services in the United Arab Emirates: A cross-sectional study. PloS one. 2021;16(6):e0253155.
85. Maselli F, Piano L, Cecchetto S, Storari L, Rossettini G, Mourad F. Direct access to physical therapy: should italy move forward? International journal of environmental research and public health. 2022;19(1):555.

86. Yang M, Bishop A, Sussex J, Roland M, Jowett S, Wilson EC. Economic evaluation of patient direct access to NHS physiotherapy services. Physiotherapy. 2021;111:40-7.
87. Demont A, Bourmaud A, Kechichian A, Desmeules F. The impact of direct access physiotherapy compared to primary care physician led usual care for patients with musculoskeletal disorders: a systematic review of the literature. Disability and rehabilitation. 2021;43(12):1637-48.
88. Pagano L, Dennis S, Wootton S, Chan AS, Zwar N, Mahadev S, et al. The effects of an innovative GP-physiotherapist partnership in improving COPD management in primary care. BMC Primary Care. 2023;24(1):142.
89. Leochico CFD, Espiritu AI, Ignacio SD, Mojica JAP. Challenges to the emergence of telerehabilitation in a developing country: a systematic review. Frontiers in neurology. 2020:1007.
90. Mohamad IS, Defi IR. Telerehabilitation in Low-and Middle-income Countries. Telehealth/Telemedicine–The Far-Reaching Medicine for Everyone and Everywhere: IntechOpen; 2022.
91. Fernandes LG, Saragiotto BT. To what extent can telerehabilitation help patients in low-and middle-income countries? Brazilian Journal of Physical Therapy. 2021;25(5):481.
92. Chamzas C, Eweje F, Kavraki LE, Chaikof EL. Human health and equity in an age of robotics and intelligent machines. NAM Perspectives. 2022.
93. de Witte L, editor Assistive Technology Provision in Low Resource Settings: Challenges and Opportunities. 2020 International Conference on Assistive and Rehabilitation Technologies (iCareTech); 2020: IEEE.
94. World Health Organization. Global Initiative on Digital Health (GIDH or 'Guide'): Executive Summary. 2023 31/July/2023.
95. World Health Organization. Global strategy on digital health 2020-2025. Geneva; 2021. Contract No.: ISBN 978-92-4-002092-4.

Supporting information

S1 Tables. Health inequity: possibility of Initiating Pulmonary Telerehabilitation Program for Adults with Chronic Obstructive Pulmonary Disorders in conflict and low-resourced area; A mixed-methods phenomenological study.

Figure-3-Triangulation model Click here to access/download;Figure;Figure-3-Triangulation-model.tiff

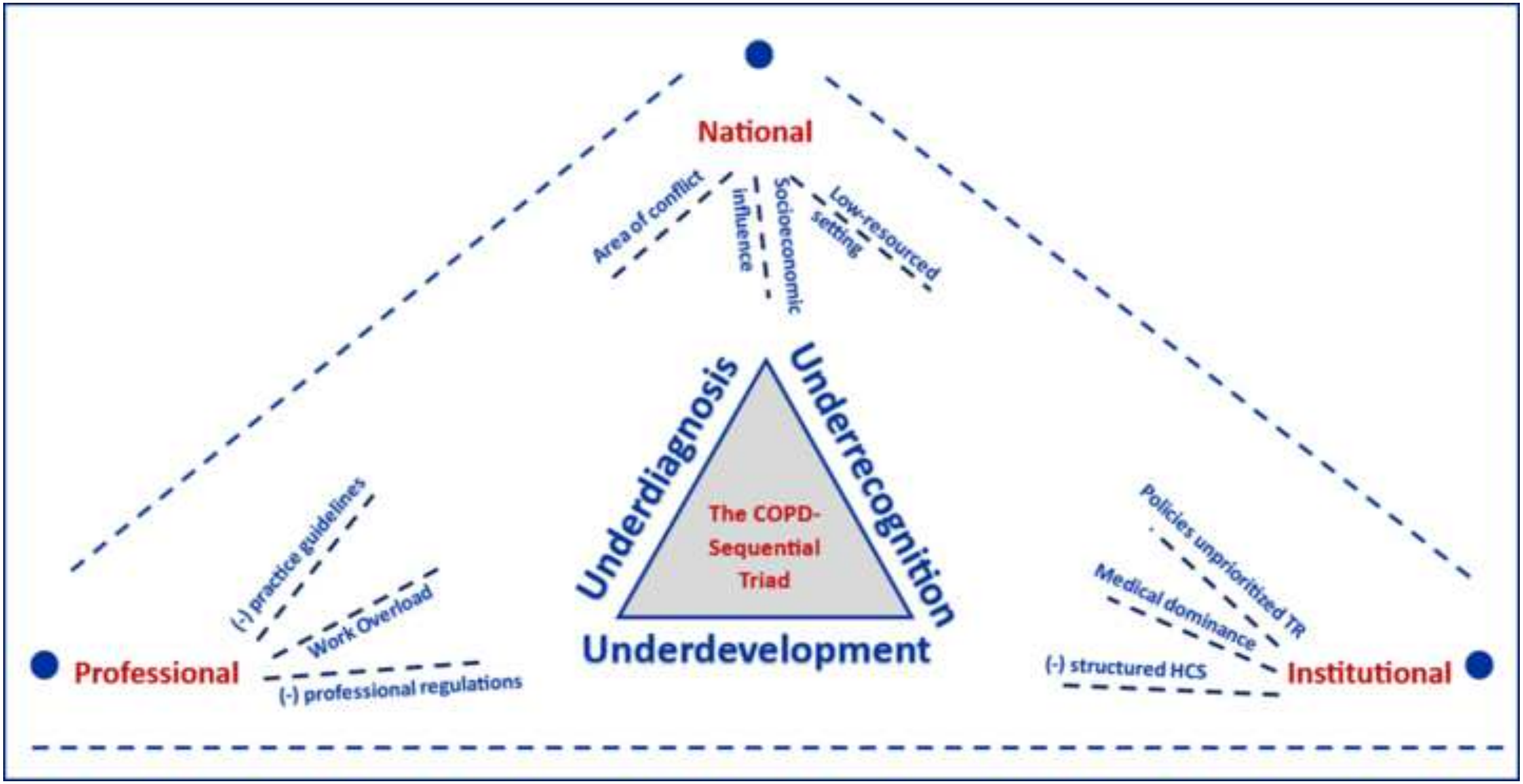

Figure-2-Thematic-Framework Click here to access/download;Figure;Figure-2-Thematic-Framework.tiff

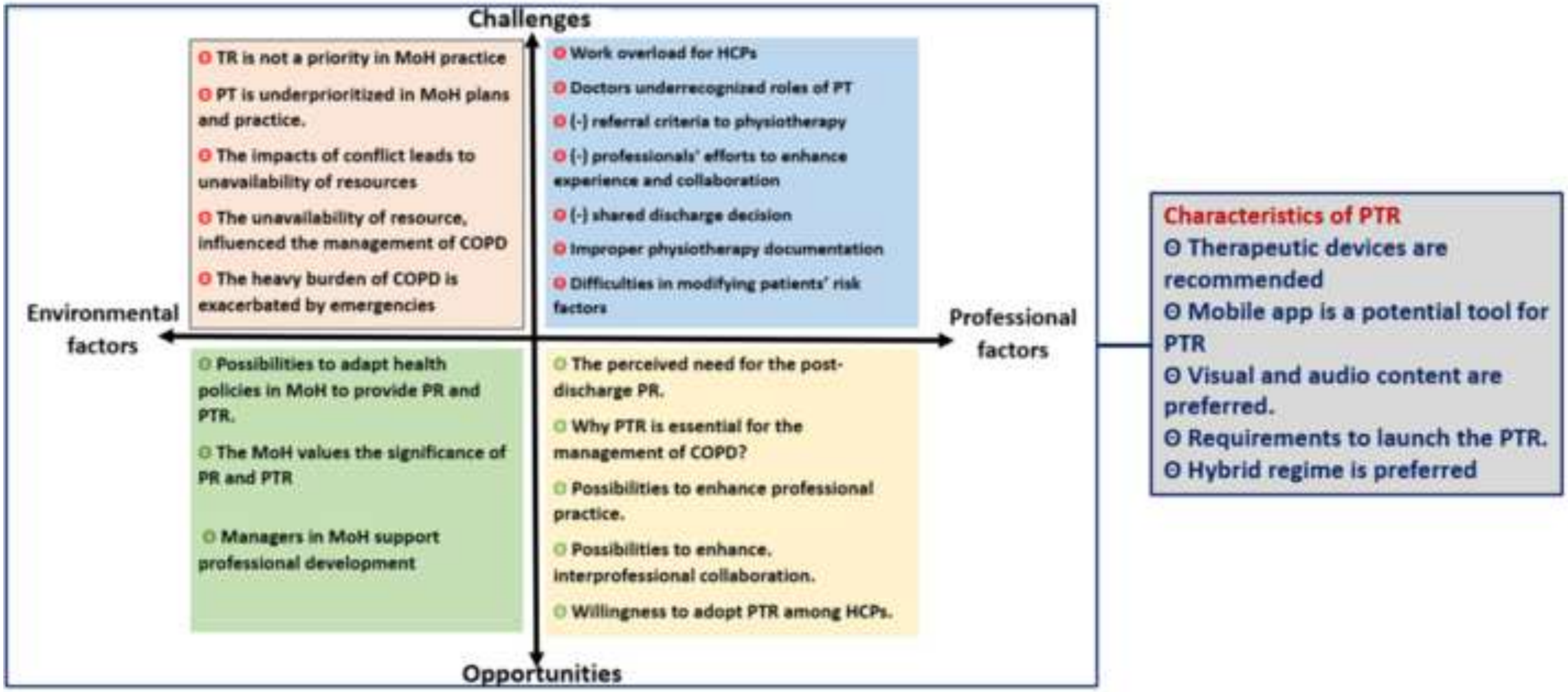

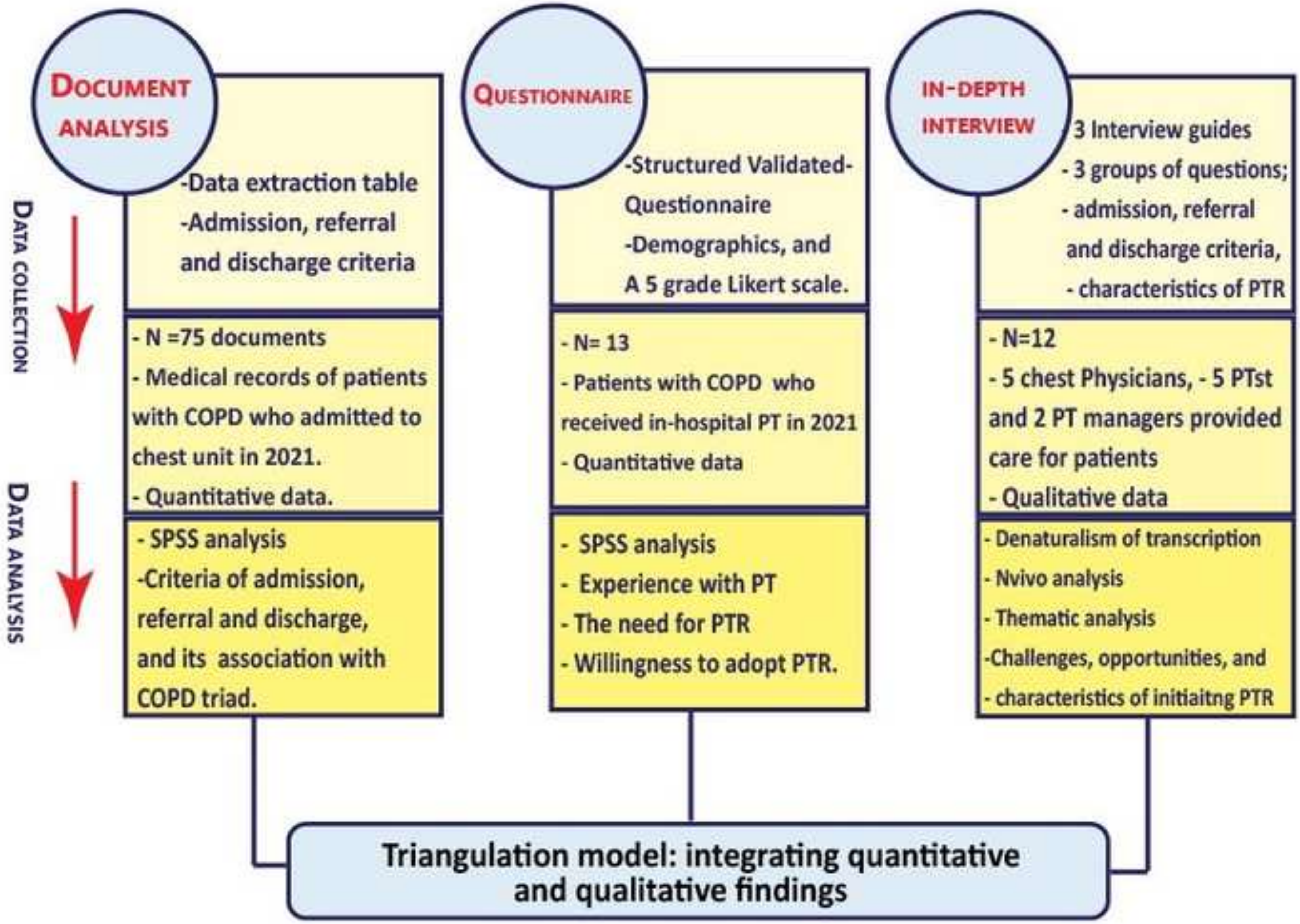
Document analysis
Questionnaire
In-depth interview
Data collection
Data analysis
-Data extraction table
-Admission, referral
and discharge criteria
-Structured Validated-
Questionnaire
-Demographics, and
A 5 grade Likert scale.
3 Interview guides
- 3 groups of questions;
- admission, referral
and discharge criteria,
- characteristics of PTR
- N =75 documents
- Medical records of patients
with COPD who admitted to
chest unit in 2021.
- Quantitative data.
- N= 13
- Patients with COPD who
received in-hospital PT in 2021
- Quantitative data
- N=12
- 5 chest Physicians, - 5 PTst
and 2 PT managers provided
care for patients
- Qualitative data
- SPSS analysis
-Criteria of admission,
referral and discharge,
and its association with
COPD triad.
- SPSS analysis
- Experience with PT
- The need for PTR
- Willingness to adopt PTR.
- Denaturalism of transcription
- Nvivo analysis
- Thematic analysis
-Challenges, opportunities, and
- characteristics of initiaitng PTR
Triangulation model: integrating quantitative
and qualitative findings

Tables of results

Click here to access/download
Supporting Information
Additional file 1.docx

Click here to access/download
Supporting Information - Compressed/ZIP File Archive
Health-inequity-conflict-LMIC.zip
