# Supplementary material for: Health inequity: Possibilities of initiating pulmonary telerehabilitation programs for adults with chronic obstructive pulmonary disorders in conflict and low-resourced areas; A mixed-method phenomenological study
Source: PLoS One. 2025 May 29;20(5):e0324624. doi: 10.1371/journal.pone.0324624 (PMC12121761; doi:10.1371/journal.pone.0324624)
Supplement: Attachment — Submitted filename: Plos One_Comments from Prof Mshunqane.docx [file pone.0324624.s007.docx]

**Comments from Prof. N. Mshunqane**

**Health Inequity; Possibilities of Initiating Pulmonary Telerehabilitation Programs for Adults with Chronic Obstructive Pulmonary Disorders in Conflict and Low-Resourced Areas; A mixed-methods Phenomenological Study**

Furthermore, If you have not already done so, we suggest that you try using another Internet browser (Editorial Manager works best with Google Chrome or Mozilla Firefox).

If the problems persist, we would be happy to submit the review on your behalf. To do so, we require a response to the following points:

1. Recommendation (Answer options: Accept, **Minor Revision**, Major Revision, Reject)

2. Is the manuscript technically sound, and do the data support the conclusions? (Answer options: **Yes**, No, Partly)

3. Has the statistical analysis been performed appropriately and rigorously? (Answer options: Yes, **No**, I don’t know, N/A):

***Qualitative phase analysis needs to be clarified. COREQ is a checklist for reporting qualitative research and not analysing data.***

4. Does the manuscript adhere to the PLOS Data Policy? Additional details can be found at [http://journals.plos.org/plosone/s/materials-and-software-sharing](https://url.za.m.mimecastprotect.com/s/VhXZC3lJV7Ck8MXksgfrHQn-mO?domain=journals.plos.org). (Answer options: **Yes**, No)

5. Is the manuscript presented in an intelligible fashion and written in standard English? (Answer options: **Yes**, No): ***This is a good scientific paper addressing the area of healthcare which addresses constrains health systems globally. This is a needed study for patients with COPD.***

6. Review Comments to the Author (minimum 200 characters):

***I would like to commend the authors for embarking*** ***in this topic and aligning with transformation in healthcare in the fourth industrial revolution where tele-health is important.***

1. **Abstract:** Please add the following: The journal starts with objective not background. Please include how qualitative data was analysed. Add key word= low resourced environment.
2. The **Background** is written well, I have a few comments which are editorial.
3. **Methods:** Please clarify if you conducted a retrospective records review or document analysis, if document analysis-provide the framework that guided the document analysis. Clarify what did the questionnaire measure, how many sections did it have? And describe its development before it was administered to participants. Please mention the language/s used when conducting interviews. How many days post hospital discharge were considered for patients to be included in the study.
4. **Data Analysis**: Quantitative data analysis is described well; however qualitative data analysis is not detailed.
5. **Results:** Triangulation of results is well described and the three themes explain the quantitative results.
6. **Discussion and conclusion:** is written well.

7. PLOS authors have the option to publish the peer review history of their article ([https://journals.plos.org/plosone/s/editorial-and-peer-review-process#loc-peer-review-history](https://url.za.m.mimecastprotect.com/s/hbqrC48KGgtVjWzVSxh1H43MxT?domain=journals.plos.org)). If published, this will include your full peer review and any attached files. Do you want your identity to be public for this peer review? (Answer options: **Yes**, No)
7a. If you answered "Yes" above, that you would like your identity to be public, please provide your full name, as it should appear on the published peer review: **Nombeko Mshunqane**

Please do not sign the review on behalf of another person.

**Confidential to Editor:**

This paper is written well, just minor editing, consistency in the use of symbols and terms. It will add value to the body of knowledge of healthcare for patients with COPD and has a potential to improve the quality of care.

Authors need to make minor corrections which involve clarifying the methodology in terms of how the questionnaire was developed? Language used and data analysis for qualitative phase. Clarify more the number of days after hospital discharge, after which participants were recruited and included in the study.

Thankyou

1. Do you have any potentially competing interests? **NONE**, If none, type "None." Our policy on competing interests can be found at [http://journals.plos.org/plosone/s/competing-interests](https://url.za.m.mimecastprotect.com/s/Cut6C580KjtJnKMJhyixHkmYDm?domain=journals.plos.org).
2. Did you receive any assistance in preparing this review (e.g. from a post-doc or graduate student)?: **NO**, If yes, please provide their name and title. If you have chosen to reveal your identity to the authors in question 7 above, please include the name of the person who assisted you in the response to question 7 as well. (optional)
3. Do you want to receive recognition for this review on a Web of Science researcher profile? ([https://clarivate.com/products/scientific-and-academic-research/research-discovery-and-workflow-solutions/researcher-profiles/](https://url.za.m.mimecastprotect.com/s/MWfvC66VKkCN39PNI5s2H5cdnm?domain=clarivate.com/))? If you opt in, your Web of Science profile will automatically be updated to show a verified record of this review in full compliance with the journal's review policy. If you don't have a Web of Science profile, you will need to first create a free account. (Answer options: **Yes**, No), *(however I do not have web of science profile, this is how I lost all the information last time, trying to create WOS profile)*.

Please do not hesitate to contact us with any additional questions. We look forward to receiving your review.

Kind regards,

Paula Katrina Maderazo
Straive Editorial Assistant

PLOS ONE | [plosone@plos.org](mailto:plosone@plos.org)
Empowering researchers to transform science

Case Number: 08778409
