# Supplementary material for: Health inequity: Possibilities of initiating pulmonary telerehabilitation programs for adults with chronic obstructive pulmonary disorders in conflict and low-resourced areas; A mixed-method phenomenological study
Source: PLoS One. 2025 May 29;20(5):e0324624. doi: 10.1371/journal.pone.0324624 (PMC12121761; doi:10.1371/journal.pone.0324624)
Supplement: Attachment — Submitted filename: Response to Reviewers-ONE-D-24-30276.docx [file pone.0324624.s009.docx]

**Dr. Suad Ghaben**

**ONE-D-24-30276 - Rebuttal letter**

**Health Inequity; Possibilities of Initiating Pulmonary Telerehabilitation Programs for Adults with Chronic Obstructive Pulmonary Disorders in Conflict and Low-Resourced Areas; A mixed-methods Phenomenological Study**

| **Comment**  **Page no. according to the PDF proof file** | **Response** | **Where?**  **Page no. according**  **to the manuscript** |
| --- | --- | --- |
| **Editorial comments\ Journal requirements** | | |
| PLOS ONE style | Aligned with PLOS ONE style |  |
| The participants provided consent for these transcripts to be published: | - A de-identified version of the data or excerpts of interview responses were uploaded to the system during submission. - Participants consented to publishing a de-identified version of the interview scripts. |  |
| A) Please clarify whether your manuscript and its Supporting Information files contain your minimal data set. The minimal data set is defined as the data set used to reach the conclusions drawn in the manuscript, with related metadata and methods, and any additional data required to replicate the reported study findings in their entirety. This may include:  a.) The values behind the means, standard deviations and other measures reported;  b.) The values used to build graphs;  c.) The points extracted from images for analysis. | - The manuscript includes the minimal data set presented in five tables, which was submitted previously as “Supporting Information”, and it is described now as S1 table, S2 table, S3 table, S4 table, and S5 table.  - We added the S1-S5 tables to the manuscript to enhance its readability.  - S1 table, and S2 table report quantitative data.  - S3 table, and S4 table, and S5 table report the qualitative data; the thematic analysis   - The minimal data (S1-S5 tables) were used to reach the conclusions, which were described under the results section and triangulation., The mean, standard deviation, and other statistical values were integrated with qualitative data to build the triangulation model and construct the study conclusion. | S1-S5 Tables  Pages 29-37 |
| If your manuscript and its Supporting Information files do not contain your minimal data set, please upload the minimal anonymized data set necessary to replicate your study findings as either Supporting Information files or to a stable public repository. | - The supporting information includes the minimal data set and is attached to this manuscript as S1-S5 Tables. | S1-S5 Tables  Pages 29-37 |
| Please amend either the abstract on the  online submission form (via Edit Submission) or the abstract in the manuscript so that they are identical. | Abstract amended as required. |  |
| review your reference list to ensure that it is complete and correct | Reference reviewed. All complete and correct |  |

| **Reviewer 1- comments** | | |
| --- | --- | --- |
| **Abstract:**  ***** The abstract should be revised for grammar and language editing. | Proofreading certificate attached |  |
| ***** The aim in the abstract is not clear. | Edited as suggested:  This study aims to explore the possibility of launching PTR programs for adults in an area of conflict and low resources. | Page 2 |
| ***** Methods not clear with regards to what has been investigated/determined. | - edited as suggested as follows:  “to generate a comprehensive understanding of the phenomena related to the unavailability of outpatient PR programs in conflict low-resourced areas, and the  possibility of launching alternative PTR programs.” | Page 6 |
| ***** What was the exact conclusion of the study? | Edited as suggested to the following:  “This study identified the national, institutional, and professional challenges and opportunities of launching PTR programs in conflict and low-resourced areas, including the conflict’s consequences, insufficient resources, and the absence of a structured healthcare system and clinical practice guidelines. Promoting health equity in such areas necessitates that the international community and institutions isolate the healthcare sector from conflicts and apply more effective strategies. The study also illustrated the characteristics of the intended PTR programs, including mHealth, therapeutic devices, and hybrid programs along with supporting  professional organizations’ guidelines.” | Page 3  Page 20 |
| **Introduction:**  ***** Line 52 – To which conflict are you referring or is this in general? | Referring to the conflict in Palestine Edited as follows:  “Crucially, the conflict in Palestine has damaged ……” | Page 3 |
| ***** Add the latest data for COPD cases and not only 2019. | The latest data related to COPD burden added:  - The sentence “It had 44,568 COPD cases and 9932 DALYs until 2019” was replaced with “The latest COPD burden for the MENA region highlighted the Age-  standardized prevalence of COPD per 100,000 population was 2000 to < 2500, the | Page 3 |

|  | number of cases was 11.42 (10.3 to 12.69) million, and the DALYs was 2.53 (2.31 to 2.8) million cases (8). And the appropriate reference cited. |  |
| --- | --- | --- |
| ***** List examples of environmental factors  limiting the access to Pulmonary rehabilitation programs. | Edited as suggested:  The following sentence was added:  “Environmental factors such as long travel distances and time, the use of public transportation, the weather, waiting time, and inappropriate health system  resources” | Page 4 |
| ***** To whom is the “others” in line 72 referring to? | Other investigators cited in references 2, 18, and 20. The following description was added:  “the Forum of International Respiratory Societies (2), and other investigators …….. | Page 4; lines 82-84 |
| ***** In general, the introduction should be revised to keep thoughts together and in a logical flow. In the current format, the facts are not following each other. | The introduction started by describing the unavailable PR programs phenomena worldwide and in conflict low-resourced areas in particular. Illustrating its administration challenges, and the possible alternatives that identified the PTR programs including therapeutic devices such as the SMART-OPEP. The situation in Palestine, as a conflict-low-resourced area was described in terms of COPD  prevalence and management including PR unavailability, and the related healthcare  system challenges were also highlighted. | Pages 3-5 |
| ***** What will the Pulmonary Telerehabilitation program consist of? | - The PTR program will consist of mHealth, therapeutic devices, a hybrid program, - Described on page 13 as follows:   “Five subthemes described characteristics of the intended PTR, as shown in Table 5 in S1 Tables; “Therapeutic devices are recommended “, Mobile app is a potential tool for PTR”, “Visual and audio contents are preferred”, “Requirements to launch  the PTR”, and “Hybrid PTR regime is preferred”. - Depicted in Figure 2   - The following sentence was added to the conclusion: - “The intended PTR program will consist of mHealth, therapeutic devices such as OPEP, and will start with a hybrid regime and be supported by professional associations guidelines.” | Page 13  Page 20-21 |

| ***** How will communication be confirmed in a conflict area in order for telecommunication? | According to this study's results; patients, physiotherapists, and doctors preferred mobile apps as a PTR tool. And it is supposed to be the mode of communication to operate the PTR program.  - This clarification was added to the end of the Results as follows: “Communication among patients and HCPs will be maintained through the mHealth app as a preferred PTR tool.” | Page 15 |
| --- | --- | --- |
| **Methodology:**  ***** The methodology is not repeatable. | - Quantitative data was collected by analyzing patients' records highlighting the medical management, referral to physiotherapy, and discharge. - Quantitative data was also collected by interview questionnaires with patients with COPD about their experience with in-hospital physiotherapy, their needs for post-discharge PR, and their willingness to adopt PTR. - Qualitative data was collected by interviewing HCPs about their experience with managing patients with COPD, providing PR, and the possibility and preferences of launching PTR. - Quantitative and qualitative data were collected in parallel, and results were triangulated to provide a comprehensive nuanced understanding of the possibility of launching PTR programs as an alternative to the unavailable PR. | Material and Methods pages 6-  10  Triangulation pages 14 & 15 |
| ***** Study process and data collection tools used are not clear. | - clarified above |  |
| ***** The aim mentioned in line 105 and 110 are not aligned. | - the aim under Material and Methods\study design was corrected to align with what was mentioned in line 105 as follows:  “ .. to generate a comprehensive understanding of the phenomena related to the unavailability of outpatient PR programs in conflict low-resourced areas, and the  possibility of launching alternative PTR programs.” | Page 6 |
| ***** To whom was the questionnaire given? | Mentioned as follows: “Patients with COPD admitted to the chest unit in 2021, received inpatient physiotherapy, and discharged from the hospital in 2021 were  included.” | Page 7; lines 164-  165 |
| ***** Who was part of the interviews? | Mentioned as follows: “We interviewed five chest physicians and five  physiotherapists who treated patients with COPD. We also interviewed two PT  managers who made clinical and administrative decisions.” | Page 8; lines 183-  186 |
| ***** How will this research inform the Smart- OPEP development? | Edited as suggested,  The following description was added at the end of the Results | End of the Results section |

|  | “Ultimately, patients and HCPs prefer using the mHealth and therapeutic devices. Therefore, the Smart-OPEP, as a PTR tool, will include mHealth and therapeutic devices such as OPEP with a preference for video and audio inclusion of the  mHealth. A hybrid regime is recommended as a start, and professional association guidelines should support the administration of PTR.” | Page 15 |
| --- | --- | --- |
| ***** Who were included for the sampling? | Patients:  “Patients with COPD admitted to the chest unit in 2021, received inpatient physiotherapy, and discharged from the hospital in 2021 were included.” | Page 7; lines 164-  165 |
|  | HCPs:  “We interviewed five chest physicians and five physiotherapists who treated patients with COPD. We also interviewed two PT managers who made clinical and administrative decisions.” | Page 8; lines 183-  186 |
|  | -documents  “We analysed medical records of patients with COPD admitted to the MoH chest unit from January 1 until December 31, 2021.” | Page 7; lines 151-  153 |
| ***** How were participants recruited? | We applied Purposive sampling according to the inclusion criteria mentioned above  “Using purposive sampling … “ was mentioned under the subheading Study Design | Page 6, line 126 |
| ***** Survey and questionnaire are used  interchangeably – be specific in which tool  was used for which objective. | Edited as suggested  The term “Questionnaire” was used throughout the manuscript and replaced all “survey” term | throughout the manuscript |
| **Results:**  ***** Suggest present the result according to the objectives. | Each objective is presented among the three study tools; which include quantitative and qualitative tools.  - Presenting the results according to objectives requires presenting quantitative and qualitative data for each of the three objectives which would confuse readers and cause inconsistency. Thus, presenting the study results according to the quantitative and qualitative tools aligned with the methodology presented, and is more convenient to facilitate the triangulation of data.  This section was included in the study's limitations |  |
| *****Data tables not visible in the document. | The tables are attached as **supporting information (S1-S5 Tables)** | S1-S5 Tables  Pages 29-37 |

|  | - **Table 1:** Results of Document analysis and demographics of the questionnaire - **Table 2:** Results of the patients' experience with the in-hospital PT, their needs for outpatient PR, and willingness to adopt PTR (2^nd^ section of the questionnaire) - **Table 3:** Thematic analysis 1-3: Challenges of launching PTR. - **Table 4:** Thematic analysis 2-3: opportunities of launching PTR. - **Table 5:** thematic analysis 3-3: characteristics of the intended PTR   programs. |  |
| --- | --- | --- |
| **Discussion & Conclusion:**  ***** The author needs to justify and interpret their findings and compare them with other  literature. | We discussed the main study results on three levels: the national, institutional, and professional levels highlighting the main challenges and opportunities for  launching PTR programs in a conflict low-resourced area. The findings were compared and contextualized with the existing literature. | Pages 16-20 |
| ***** The novelty of the study is not clear in the discussion. | The novelty of the study was highlighted in a paragraph added to the discussion section and appropriate citations were added, as follows:  - “.. This work also emphasized the possibilities of the transformation to telehealth even in conflict-low-resourced areas, which aligns with the fourth industrial revolution (60). By tackling the identified challenges and seizing the available opportunities, such novelty can be achieved. Launching PTR programs also requires the allocation of resources, working in MDTs, and aligning with the appropriate  frameworks and models (26, 61).” | Page 16 |
| **General:**  Suggestion that the manuscript be submitted for language and grammar editing.  The manuscript should be written in scientific  language and be more concise. | The transcript was revised for scientific concise writing and language grammar editing.  Proofreading certificate attached |  |
| **Reviewer 2- comments** | | |
| Thankyou for the opportunity to review this informative work. I would like to commend  the authors for embarking in this topic and |  |  |

| aligning with transformation in healthcare in the fourth industrial revolution where  telehealth is important. |  |  |
| --- | --- | --- |
| **Abstract:**  Please add the following:  ***** The journal starts with objective not background. | Edited as suggested:  “Objective: The "triple problems of COPD"—underdiagnosis, underrecognition, and underdevelopment of pulmonary rehabilitation programs—are global. Pulmonary telerehabilitation (PTR) may solve these problems. This study aims to explore the possibility of launching PTR programs for adults in an area of conflict and low  resources. | Page 2 |
| ***** Please include how qualitative data was analysed. | Edited as suggested.  The following description was added to the method section of the abstract: “Quantitative data was analyzed descriptively, and inferentially by the SPSS, outlining the means, standard deviations, percentages, the chi-square, respectively.  Qualitative data was analyzed thematically using the NVivo software, and five themes and 20 subthemes were identified. A thematic framework and  triangulation model were depicted accordingly.” | Page 2 |
| ***** Add key word= low resourced environment | Edited as suggested:  Low-resourced area was added to the keywords | Page 3 |
| **The Background** is written well, I have a few comments which are editorial. | All typos such as] were corrected. | Refer to the attached PDF for  comments |
|  | **Are you using a spirometer or Spirometry? to assess lung disorder such as COPD?** Spirometry is the test of measuring lung volume, and the spirometer is the device used for this purpose; therefore, spirometry was used when it describes the lung  volume measure, and spirometer was used when it describes the device used. | Page 4, 5,  Page 10 |
|  | Start a sentence with a full term, not an abbreviation. or start with "The" Edited as suggested | Page 5 |
| **3. Methods:**  ***** Please clarify if you conducted a  retrospective records review or document | We conducted a retrospective records analysis;  We implemented the medical management framework as described in GOLD. | Page 7: lines 151-  153 |

| analysis, if document analysis, provide the  framework that guided the document analysis. | The following description was added “We analysed medical records (48) of patients with COPD admitted to the MoH chest unit from January 1 until December 31,  2021. We applied the management framework as described by GOLD (1).” |  |
| --- | --- | --- |
| *****Do you mean patients records review? If yes, records review is different from document analysis.  *****Analysis is done to interpret document and gain their meaning whilst review is to identify patterns of healthcare, see Kayesa  and Shung-King, 202 | Records analysis – as explained above .. and the mentioned literature was cited. | Page 7 |
| - was the questionnaire developed from the draft done by SG? - If so which components of the records were considered for the questionnaire and which ones formed an interview guide? | The questionnaire was developed from the draft prepared by SG.  -The preliminary interview and the preliminary analysis of medical records, both informed the development of the three data collection tools.  -The components of the records have not specifically informed the development of the questionnaire or the interview guide.  -The following sentence was added to the description:  “Based on the preliminary interview results, data from medical records, and  literature search, SG prepared the draft version of the three study tools, which were discussed thoroughly among the team (SG, BKH, RK, MH, AFML, and DKAS) and further refined.” | Page 6; lines 140-  143 |
| ***** Clarify what did the questionnaire measure, how many sections did it have? And describe its development before it was administered to participants. | -In addition to the SD section, it measures the patients' experience with in-hospital PT, the need for outpatient PR, and their willingness to adopt PTR.  -Before administration, the questionnaire was validated for face and content validity by three physiotherapy researchers.  -A detailed description of the questionnaire is provided on page 7 under the  subheading: Questionnaire | Page 7; lines 143-  147 |
| what did this questionnaire measure? e.g Knowledge or a specific process?  Was the questionnaire developed in a specific language, e,g a native or formal language? | - The questionnaire measured the patients' experience with in-hospital   physiotherapy, their need for post-discharge PR, and their willingness to adopt PTR.   - The questionnaire was developed in the formal Arabic language. | Page 8; lines 168-  170  Page 8; line 166-  167 |

| What does PT stand for? is it Pulmonary therapy? | PT stands for Physiotherapy  It was described when first mentioned under the results section of the abstract, and document analysis under the data collection. | Page 2  Page 7 |
| --- | --- | --- |
| was the questionnaire developed in Arabic? | Yes.  The following sentence was added:  “The questionnaire was developed in Arabic based on the preliminary interviews and the medical records.” | Page 7 |
| Is this the same questionnaire that was developed in this study? | Yes  We applied one questionnaire: The first section includes the demographic data, and the second section includes 27 items. It was developed, validated, reliability tested, and then administered. | Page 8 |
| the process of development needs to be  clarified and reported in a coherent manner: e,g   1. development 2. language 3. sections and items 4. validation and reliabilty, finally, 5. how or for what was it used in this study? | Edited as suggested | Page 7 & 8 |
| “The Arabic questionnaire was translated into English for review and verification by AFML and DKAS”  This part must go up and be clarified before administering the tool, describe it first then clarify how it was applied. | Edited as suggested | Page 8 |
| Qualitative phase analysis needs to be  clarified. **COREQ** is a checklist for reporting qualitative research and not analysing data. | The QOREQ checklist was used for data reporting rather than data analysis, as clarified in the following description which was included in the text:  “Findings were reported according to the Consolidated Criteria for Reporting Qualitative Research (COREQ)” | Page 9  Under data analysis  - Qualitative data |

| Who are PT managers? | Edited as suggested, the following was added to the text:  -“including the head of the Physiotherapy department at the hospital and the general manager of the Physiotherapy and Rehabilitation unit at the MoH.” | Page 8 |
| --- | --- | --- |
| ***** Please mention the language/s used when conducting interviews. | Edited as suggested:  The following sentence was added:  “Interviews were conducted and in Arabic, allowing participants to  openly express their viewpoints. | Page 9 Under the  qualitative data subheading |
| ***** How did researchers adhere to trustworthiness? | Trustworthiness was attained in the mentioned section on page 9 & 10 “Participants’ trust and rapport, which were built through prolonged engagement with the research team, helped sustain credibility.”  “Member checking and triangulation verified thematic analysis; participants’ trust and rapport were attained through prolonged engagement; researchers and HCPs were involved for years in variable projects.” | Page 9 |
| “13 patients with COPD admitted to the chest unit and received in-hospital PT completed the questionnaire.”  ***** How many days post-hospital discharge was considered for patients to be included in the study. | -The post-hospital discharge time was not considered as a factor. We included patients who received in-hospital PT in 2021 regardless of the number of days post- discharge. The number of patients who received in-hospital PT was 17 and was distributed throughout 2021 from January to December.  The relevant text was amended to:  “Patients with COPD admitted to the chest unit in 2021, who received in-hospital PT and were discharged from the hospital in 2021, regardless of the number of days post-discharge, were included. | Page 7,8 |
| **5. Results:** The triangulation of results is well described and the three themes explain the  quantitative results. | Thanks |  |
| **Patients valued post-discharged PT**  how often did they come post-discharge? | The hospital does not provide post-hospital PR as a routine practice. It depends totally on the physiotherapist-patient relationship and the willingness of the physiotherapist to keep post-discharge communication with patients.  The text was amended to:  “Although post-discharge PT was not a routine practice in the hospital, it was considered valuable by patients, …..” | Page 12 |

| **6. Discussion and conclusion:** is written well. | Thanks |  |
| --- | --- | --- |
| **General:**  *****This paper is written well, just minor editing, consistency in the use of symbols and terms.  *****It will add value to the body of knowledge of healthcare for patients with COPD and has a potential to improve the quality of care.  *****Authors need to make minor corrections which involve clarifying  *****the methodology in terms of how the questionnaire was developed?  *****Language used and data analysis for qualitative phase.  *****Clarify more the number of days after  hospital discharge, after which participants were recruited and included in the study. | Edited as suggested |  |

Our reference: **CERT-180225-03Suad**

***Certificate of Acknowledgement (Proofreading)***

# We certify that the following Journal Article (MS Word)

Health Inequity: Possibilities of Initiating Pulmonary Telerehabilitation Programs for Adults with Chronic Obstructive Pulmonary Disorders in Conflict and Low- Resourced Areas; A Mixed-method Phenomenological Study

# by

**Dr. Suad Ghaben**

has undergone the Proofreading service from Proofreaders United.

Document length: 17.5 page(s) based on our format (Times New Roman, font size 12, 2.0 line spacing, without figures and tables).

Full details of the Proofreading service can be found at [ProofreadersUnited.com.](https://proofreadersunited.com/)

24 February 2025 Acknowledgement by:

**DR. MUHAMMAD ZAKI RAMLI FOUNDER OF PROOFREADERS UNITED**
